# The Burdens of Idiopathic Developmental Intellectual Disability Attributable to Lead Exposure from 1990 to 2023 and a Projection to 2050 in the USA: Findings from the Global Burden of Disease Study 2023

**DOI:** 10.3390/healthcare14040508

**Published:** 2026-02-16

**Authors:** Bingyan Hu, Jeryl D. Benson

**Affiliations:** John G. Rangos, Sr. School of Health Sciences, Duquesne University, Pittsburgh, PA 15282, USA; hub@duq.edu

**Keywords:** intellectual disability, idiopathic developmental intellectual disability, lead exposure, USA, disability-adjusted life years, GBD 2023

## Abstract

**Background**: Idiopathic developmental intellectual disability (IDID) is a neurodevelopmental disorder that leads to poor health status. This study analyzes the burden of IDID attributed to lead (Pb) exposure in the United States of America (USA) from 1990 to 2023 and projects trends through 2050. **Methods:** Measurements on Disability-adjusted life years (DALYs) and Years lived with disability (YLDs) were downloaded from the Institute for Health Metrics and Evaluation (IHME). A joinpoint regression model was employed to assess the epidemiological change in this disease. The age–period–cohort (APC) model was used to examine the age, period, and birth cohort effects on DALYs. Decomposition analysis was applied to analyze the role of population, aging, and epidemiological factors in driving changes to DALYs. Bayesian age–period–cohort (BAPC) analysis was conducted to forecast sex-specific burden trends through 2050. **Results:** From 1990 to 2023, DALYs and age-standardized DALY rate (ASDR) showed an overall decreasing trend. Males bore a higher disease burden than females. In the USA, the average annual percentage change (AAPC) in ASDR was −1.41 (95% CI: −1.45 to −1.37), indicating an overall decline. BAPC analysis predicted that the ASDR will continue to decline for both females and males through 2050, with males showing a faster decline. **Conclusions:** Consistent efforts have led to significant progress in reducing lead exposure-related IDID in the USA. Prevention strategies focus on continuing to reduce lead exposure and minimize its impact on IDID.

## 1. Introduction

Intellectual disability (ID) is a neurodevelopmental disorder. Intellectual functioning is assessed on the basis of the intelligent quotient (IQ), a total score based on standardized intelligence tests. The IQ test score is standardized to a median of 100 and a standard deviation of 15, with scores of 70 or below (two standard deviations below the median) suggesting intellectual limitations [1]. According to Diagnostic and Statistical Manual of Mental Disorders, Fifth Edition (DSM-V), severity was specified according to mild, moderate, severe, and profound [2] (pp. 34–37). Idiopathic developmental intellectual disability (IDID) is the category of intellectual disability cases of unknown origin after all other sources of intellectual disability are excluded, which constitutes about half of all intellectual disability cases [3,4]. Individuals with ID experience poorer health status than the general population. Dysfunctions for IDID include, but are not limited to, intellectual functioning, cognition, adaptive behavior, emotional regulation, and interpersonal relationship [5,6].

The burden of IDID is attributable to a variety of risk factors, including maternal mental illness, maternal infections, birth complications, and socio-environmental influences [7]. Among the broad etiological landscape, lead exposure was a major risk factor for IDID [7,8]. According to the Global Burden of Disease (GBD) 2023 estimates, idiopathic developmental intellectual disability attributable to lead exposure accounted for approximately 0.13% of total global Disability-adjusted life years (DALYs) (95% UI: 0.064–0.24%), with about 61.53% (95% UI: 31.24–79.83%) of the burden attributable to lead exposure. In the United States, the burden was lower, with idiopathic developmental intellectual disability accounting for only 0.092% of total DALYs (95% UI: 0.043–0.18%), and an estimated 46.23% (95% UI: 20.83–66.4%) of this burden attributable to lead exposure. Specifically, the United States experienced 15.26 DALYs per 100,000 population for this condition. Lead exposure likely impacts the function of multiple organ systems, particularly the nervous, skeletal, and cardiovascular system, resulting in fine motor and emotional regulation deficits with important outcomes. Lead exposure causes cognitive impairment during the lifespan. Blood lead concentrations show a negative correlation with intellectual functions, as reflected by low IQ scores [3,9,10]. Beyond its health effects, lead poisoning carries diverse social and economic consequences, including higher healthcare spending, loss of productivity and income, increased need for special education services, associations with behavioral problems, and lower socioeconomic status in later life [11,12].

However, few studies have evaluated the burden of IDID attributable to lead exposure at either the global or national levels. Studies that assess the corresponding epidemiological data in the USA and how this burden has evolved over time across different locations is lacking. This study aims to examine the burden and spatiotemporal pattern in the United States, as well as subnational trends across the 50 states and the District of Columbia. Using the data source of the GBD study (2023), the aim of this paper is to fill the knowledge gap of the disease burden of lead exposure-associated IDID in the United States of America and the District of Columbia. The findings are expected to offer guidance for developing targeted strategies to reduce the impact of lead exposure on IDID and improve related health outcomes in the USA.

## 2. Materials and Methods

### 2.1. Data Collection

The GBD 2023 database was used to extract data on IDID attributable to lead exposure in the United States from 1990 to 2023. All the data for the United States IDID burden attributable to lead exposure were downloaded from the Institute for Health Metrics and Evaluation (IHME). The detailed screening criteria were as follows: (1), GBD estimate was selected as a “risk factor”; (2), the locations were selected as “United States of America”, which included all 50 states and the District of Columbia; (3), the cause was specified as “idiopathic developmental intellectual disability”, and the risk selection was “lead exposure”; (4), the measures were “DALYs” and “YLDs”; and (5), years from 1990 to 2023 were included on an annual basis. We sourced the GBD population estimates of IDID attributable to lead exposure (1990–2023) from the Global Health Data Exchange (GHDx) platform. In addition to the GBD population data of IDID attributable to lead exposure spanning from 1990 to 2023, we retrieved GBD population forecasts from 2024 to 2050 in the United States of America from the Global Health Data Exchange (GHDx) website. In the GBD comparative risk assessment framework, lead-attributable burden is estimated using population attributable fractions (PAFs) based on each exposure–response relationship and a theoretical minimum risk exposure level (TMREL) [13]. As this study is based on GBD-modeled estimates, uncertainties are inevitably introduced due to limitations in input data and the assumptions underlying the exposure–response relationships [14].

### 2.2. Indicators of Disease Burden

In this study, Disability-adjusted life years (DALYs) were analyzed by location, gender, and age. DALYs represent the sum of years of life lost due to premature death and years lived with disability. DALYs were calculated by summing years lived with disability (YLDs) and years of life lost (YLLs). The estimated YLLs for mental disorders were extremely low and fail to reflect premature mortality in people affected by mental disorders, such as idiopathic intellectual disability. IDID is a non-fatal disease; the number of YLLs for premature death is 0, i.e., DALYs = YLDs. When comparing the burden of IDID in different age groups or at different periods in the same population, we used the age-standardized rate (ASR) to report per 100,000 population, with the respective 95% uncertainty intervals (UIs) from the GBD database. The 95% UIs were defined as the 2.5th and 97.5th percentiles of the posterior distribution of the model estimates. This approach accounts for differences in age structure and allows valid comparisons over time and between groups, which was calculated using the world standard population [15,16]. Percent change was used to quantify the relative difference between DALYs in 2023 and 1990. In this study, the age-standardized DALY rate (ASDR) was calculated to account for variations in age structure and facilitate consistent comparisons across populations based on the world standard population age structure [17]. The formula for calculating ASDR for IDID caused by lead exposure is outlined below:

(1) $$ASDR=\frac{\sum_{i=1}^{A}a_{i}w_{i}}{\sum_{i=1}^{A}w_{i}}\times 100,000$$

In this formula, *α_i_* represents the age-specific rate in the *i*-th age group (per 100,000 population), *w_i_* indicates the number of people in the *i*-th corresponding age group among the standard population and *A* is the number of age groups.

Annual percentage change was calculated to quantify the year-to-year rate of change within the specific time frame. The average annual percentage change (AAPC) was used to measure the trend of ASDR over the entire study period, calculated as a weighted average of segment-specific annual percentage change [18]. In this study, the time frame for annual percentage change and AAPC were assessed over the study period of 1990 to 2023.

Using R software (version 4.5.1), combined plots were generated to present the temporal trends for both the DALY number and ASDR for lead-related IDID for females and males. DALYs are exhibited through bar charts with 95% UIs shown as black vertical error bars, while ASDR is displayed as line charts with 95% UIs shown as shaded ribbons. Pyramid plots were generated illustrating DALYs by age group and sex for 1990 and 2023, with bars extending to the left for females and to the right for males. Corresponding line charts were generated to represent the age-specific DALY rate (per 100,000 population) for the same years, with shading indicating 95% UIs. To illustrate geographic disparity of DALYs and ASDR, and temporal trend changes across U.S. states and the District of Columbia, choropleth maps were generated using https://www.bioinformatics.com.cn (accessed on 20 November 2025), a digital platform for analyzing and visualizing data [19]. Subnational values are displayed with sequential color gradients to represent the magnitude of each metric.

### 2.3. Joinpoint Regression Model

To evaluate the valuation of trends in the ASDR of IDID caused by lead exposure during 1990–2023 in the United States, joinpoint regression analysis was applied. The joinpoint regression model (version 5.4.0.0, National Cancer Institute, USA; https://surveillance.cancer.gov/joinpoint/download, accessed on 6 November 2025) fits a series of linear segments to assess temporal trends in disease burden [20]. This approach applies piecewise regression to describe changes in trends over time by segmenting the study period into distinct intervals. Within each interval, trend lines are fitted and optimized, allowing for observation of how disease burden evolved across regions over the entire study period [21]. The optimal number of significant joinpoints was selected using permutation tests. *p*-values were estimated using Monte Carlo procedures, with the overall asymptotic significance level controlled by Bonferroni correction [22,23]. In the joinpoint regression model, each characterized by a slope that represents the annual percentage change for that period. A log-linear model was employed to fit the DALYs, ln(*y*) = *α* + *βx* + *ε*, where y is the ASDR of lead exposure-induced IDID, *α* the constant, *β* the slope coefficient, and *ε* the random error. For each segment defined by joinpoints, the annual percentage change was calculated using the formula

(2) $$Annual\;percentage\;change=\left(e^{\beta }-1\right)\times 100$$

To summarize long-term trends, the average annual percentage change (AAPC) was estimated and its 95% confidence interval (CI) derived from the combination of multiple annual percentage changes [24]. Statistical significance was defined as a *p* value less than 0.05, indicating meaningful epidemiological trends. When the AAPC > 0 with a *p* value < 0.05, the age-standardized rates demonstrate a statistically significant increasing trend over the study period; conversely, when the AAPC is <0 with a *p* value < 0.05, the age-standardized rates indicate a statistically significant decreasing trend. Consistently, the trend was also considered upward (or downward) if both the AAPC and its 95% CI were above zero (or below zero) and stable if the 95% CI included zero [25,26]. Based on data from GBD 2023, the fluctuation trend at the national level was statistically significant as we compared AAPC to 0. Change in the DALY number was reported as percentage change (the difference between the value in 2023 and the value in 1990 divided by the value in 1990). The AAPC was computed using the general formula

(3) $$AAPC=\left\{\exp\left(\frac{\sum w_{i}b_{i}}{\sum w_{i}}\right)-1\right\}\times 100$$

where *b_i_* denotes the slope coefficient for the *i*-th segment within the selected range of years and *w_i_* represents the number of years included in that segment.

### 2.4. Age–Period–Cohort Model

The age–period–cohort model was utilized to examine the age, period, and birth cohort effects on DALYs for lead exposure-induced IDID from 1990 to 2023. This model outperforms traditional epidemiological methods in attributing the contribution of age-related biological factors as well as technological and social factors to the underlying trend of disease burden [27]. The age effects refer to differences in disease DALY rates among different age groups; the period effects represent temporal changes in disease DALY rates in populations; and the cohort effects reflect generational differences in disease DALY rates exposed to varying risk factors among birth cohorts [28]. The net drift represents the overall annual percentage change, while the local drift reflects the annual percentage change in the ASDR within each specific age group [29].

### 2.5. Decomposition Analyses

The Das Gupta method of decomposition analysis is an epidemiological technique that attributes changes in disease burden to three contributing factors: population growth, population aging, and epidemiological changes. Decomposition analysis was conducted to demonstrate the extent to which changes in DALYs were driven by each factor over the study period. The proportions of DALYs attributable to these three factors for lead exposure-related IDID in the USA between 1990 and 2023, as well as for each region included in this analysis, were presented. Positive values indicate that the factor contributed to an increase in DALYs, while negative values indicate a reduction [30].

### 2.6. Bayesian Age–Period–Cohort (BAPC) Model

Using a Bayesian approach, projected trends were expressed probabilistically based on historical data [31,32]. GBD data was used from 1990 to 2023 to forecast the burden of IDID attributable to lead exposure in the United States of America from 2024 to 2050 by employing the BAPC model. The trends of both American males and females were forecasted. This model was implemented using the Integrated Nested Laplace Approximation (INLA) and Bayesian age–period–cohort (BAPC) packages in R to forecast IDID DALYs attributable to lead exposure through 2050 [33,34]. To assess the predictive accuracy of the BAPC model, the model was fitted using data from 1990 to 2018 and used to predict values for 2019–2023. Predicted values were then compared with the observed data for the same period. Predictive performance was evaluated using the root mean square error (RMSE) between observed and predicted values. A lower RMSE indicates better predictive performance [32]. The model demonstrates flexibility and robustness in analyzing time series data and is therefore suitable for long-term projections for disease burden. Given its comprehensive framework and ability to characterize temporal patterns, the BAPC model has been extensively validated and adopted in epidemiological research, particularly in analyses incorporating age-structured population and complex cohort effects [35].

## 3. Results

### 3.1. Burden of IDID Attributable to Lead Exposure in the USA and Across USA States

**Figure 1:**
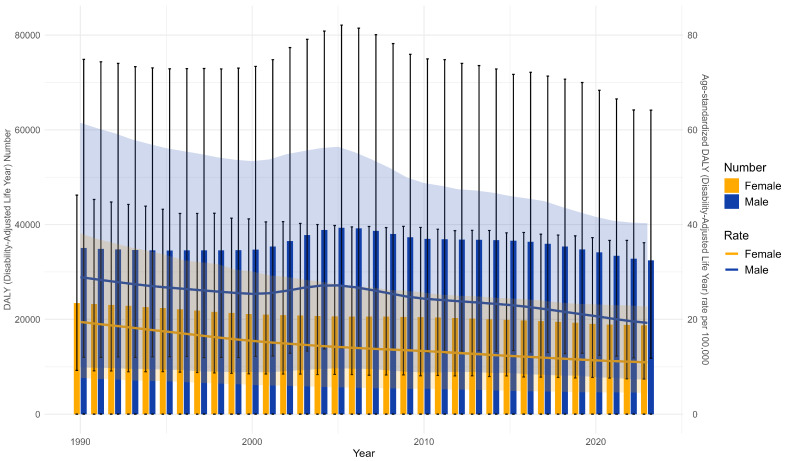
The DALY number and ASDR of IDID attributable to lead exposure in the USA from 1990 to 2023.

This study systematically assessed the trends in disease burden attributable to lead exposure-induced IDID in the United States from 1990 to 2023 (Table 1; Table S1; Figure 1). Table 1 summarizes national estimates for females and males, while detailed state-level results are shown in Supplementary Table S1. From 1990 to 2023, the DALYs and ASDR for this disease burden showed a decreasing trend in the United States of America. In 1990, the number of IDID-associated DALYs caused by lead exposure was 58,508 (95% UI: 21,112–122,048) and decreased to 51,194 (95% UI: 19,265–98,846) in 2023, reflecting a 12.50% decline. In 1990, the ASDR was 24.21 (95% UI: 8.8–50.25) per 100,000 and declined to 15.14 (95% UI: 5.89–31.5) per 100,000. The AAPC in ASDR was −1.41 (95% CI: −1.45 to −1.37). Males bore a higher burden compared to females in the USA over this study period, as shown by the number of DALYs and ASDR per 100,000 population (Table 1 and Figure 1). Between 1990 and 2023, the DALY number for American males decreased from 35,073 (95% UI: 12,018–74,896) to 32,444 (95% UI: 11,827–64,166), representing a 7.50% reduction. The DALY number for American females decreased from 14,680 (95% UI: 4883–28,459) to 18,750 (95% UI: 7437–36,177), representing a 19.99% reduction. The ASDR for American males changed from 28.91 (95% UI: 9.9–61.5) per 100,000 to 19.26 (95% UI: 7.31–40.29) per 100,000. The ASDR for American females changed from 19.45 (95% UI: 7.68–38.15) per 100,000 to 10.97 (95% UI: 4.47–22.7) per 100,000.

**Figure 2:**
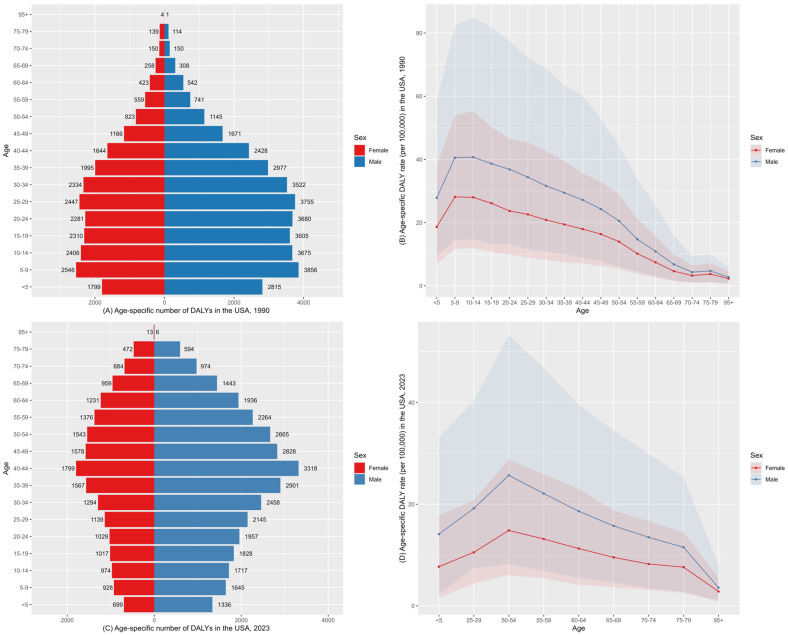
Age-specific DALY numbers and rates by sex in 1990 and 2023 in the USA. (**A**) Age-specific number of DALYs in 1990; (**B**) age-specific DALY rate (per 100,000) in 1990; (**C**) age-specific number of DALYs in 2023; (**D**) age-specific DALY rate (per 100,000) in 2023.

States such as Montana and Wyoming showed the steepest declines in AAPC, whereas states including North Carolina and Idaho experienced increasing DALYs despite a declining ASDR. Figure 1 shows the ASDR by sex in the USA from 1990 to 2023. The ASDR in males remained consistently higher than that in females throughout the study period. In both 1990 and 2023, the difference in age-specific DALY rate (per 100,000 population) narrowed above age 55 years between males and females. The age-specific DALY rate (per 100,000 population) increased from the 50–54 age group onward for males and females. During this period, males demonstrated a faster rate of decrease than females (Figure 2; Table S2).

**Figure 3:**
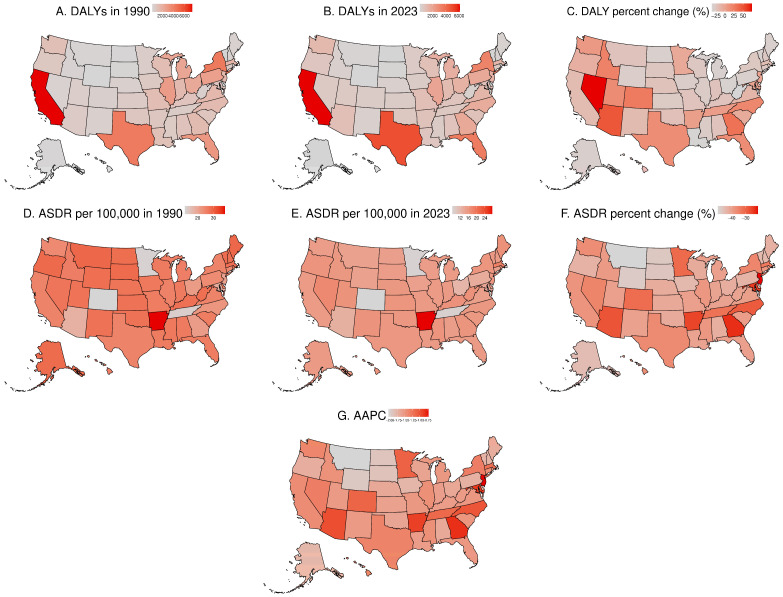
(**A**) DALY number in 1990; (**B**) DALY number in 2023; (**C**) percentage change in DALY number from 1990 to 2023; (**D**) ASDR in 1990; (**E**) ASDR in 2023; (**F**) percentage change in ASDR from 1990 to 2023; (**G**) AAPC in ASDR from 1990 to 2023.

The results for DALYs and ASDR per 100,000 across the U.S. are presented in Figure 3. Across the 50 U.S. states and the District of Columbia, the highest number of DALYs was seen in California. In contrast, the lowest number was observed in Wyoming both in 1990 and 2023 (Figure 3A,B; Table S1). In 2023, the state with the highest ASDR per 100,000 population was Arkansas. The state with the lowest ASDR per 100,000 population was Colorado (Figure 3E; Table S1).

### 3.2. Joinpoint Regression Analysis

**Figure 4:**
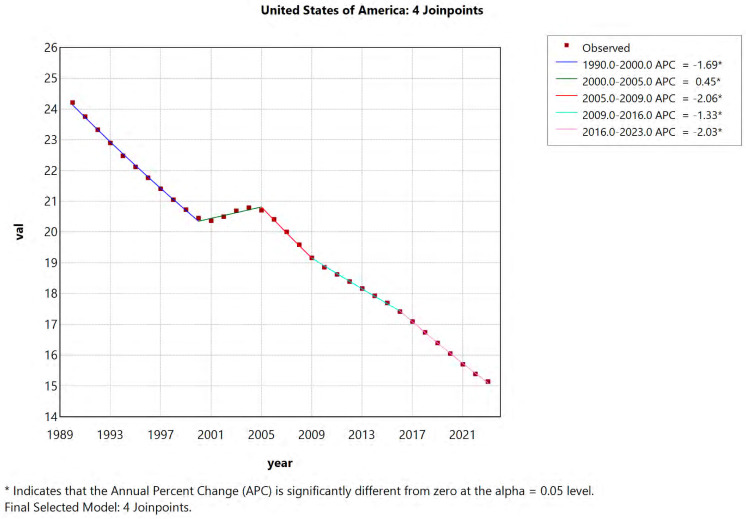
Joinpoint analysis of IDID attributable to lead exposure in the USA from 1990–2023.

Figure 4 presents joinpoint regression analyses of the ASDR per 100,000 population in the USA from 1990 to 2023. Also, we conducted analysis for all 50 states and the District of Columbia over the study period (Figures S1–S51). Joinpoint regression analysis revealed overall decreasing trends in ASDR across all locations from 1990 to 2023. At the national level, DALYs in the USA declined over time with an upward trend between 2000 and 2005 and downward trends in the remaining time segments. This pattern was also observed in most states. Arizona, Arkansas, Colorado, Maryland, New Jersey, North Carolina, and Tennessee showed consistently decreasing DALYs throughout the study period. There was a difference in the magnitude of decrease in ASDR, with AAPC ranging from −0.82% in Georgia to −2.05% in Montana. If the AAPC is negative and its 95% confidence interval lies entirely below 0, the downward trend is considered statistically significant. AAPCs are provided in Table S1 and Figure 3G.

### 3.3. Age–Period–Cohort (APC) Model Estimation of IDID Trends Attributable to Lead Exposure in the USA

**Figure 5:**
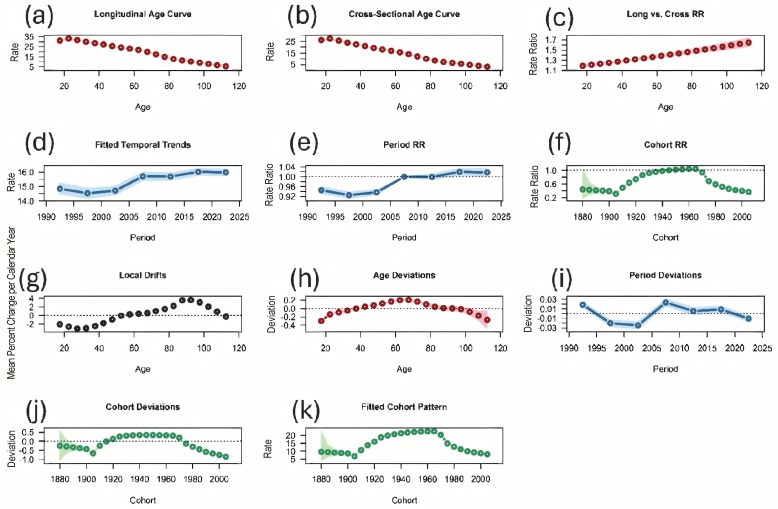
Age–Period–Cohort (APC) analysis of DALYs for IDID attributable to lead exposure in the USA. (**a**) Longitudinal age curve; (**b**) cross-sectional age curve; (**c**) longitudinal vs. cross-sectional rate ratios (RRs); (**d**) fitted temporal trends; (**e**) period rate ratios (RRs); (**f**) cohort rate ratios (RRs); (**g**) local drifts (age-specific annual percentage change); (**h**) age deviations; (**i**) period deviations; (**j**) cohort deviations; (**k**) fitted cohort pattern.

The APC model was used to estimate the age, period, and cohort effects on the DALYs for IDID attributable to lead exposure in the USA (Figure 5). The APC model showed a significant upward temporal trend, with a net drift of 0.293% per year (95% CI 0.203 to 0.384). Although the APC model yielded a positive net drift, this does not conflict with the declining ASDR trend. Net drift represents the underlying temporal change after accounting for age effects, whereas ASDR summarizes the observed pattern in age-standardized rates. Accordingly, these measures describe different dimensions of temporal change and are not directly comparable. Age, period, and cohort deviations were all significant (*p* < 0.05), indicating heterogeneous age-specific risks, time-specific influences, and substantial generational differences. Local drift estimates varied across age groups and differed from the overall net drift (*p* < 0.05), suggesting non-uniform annual changes. The longitudinal age trend (LAT) was –0.018, and the cohort age trend (CAT) was –0.021, indicating decreasing log–rates with increasing age and lower underlying risk in more recent birth cohorts (Table S3).

### 3.4. Decomposition Analysis of IDID Trends Attributable to Lead Exposure in USA and Across States

**Figure 6:**
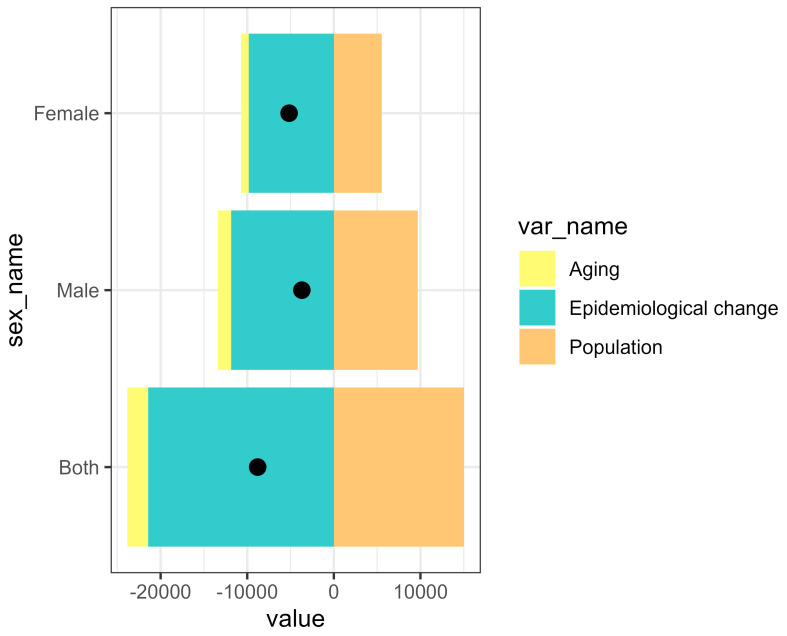
Decomposition analysis of DALYs of IDID attributable to lead exposure in the USA.

**Figure 7:**
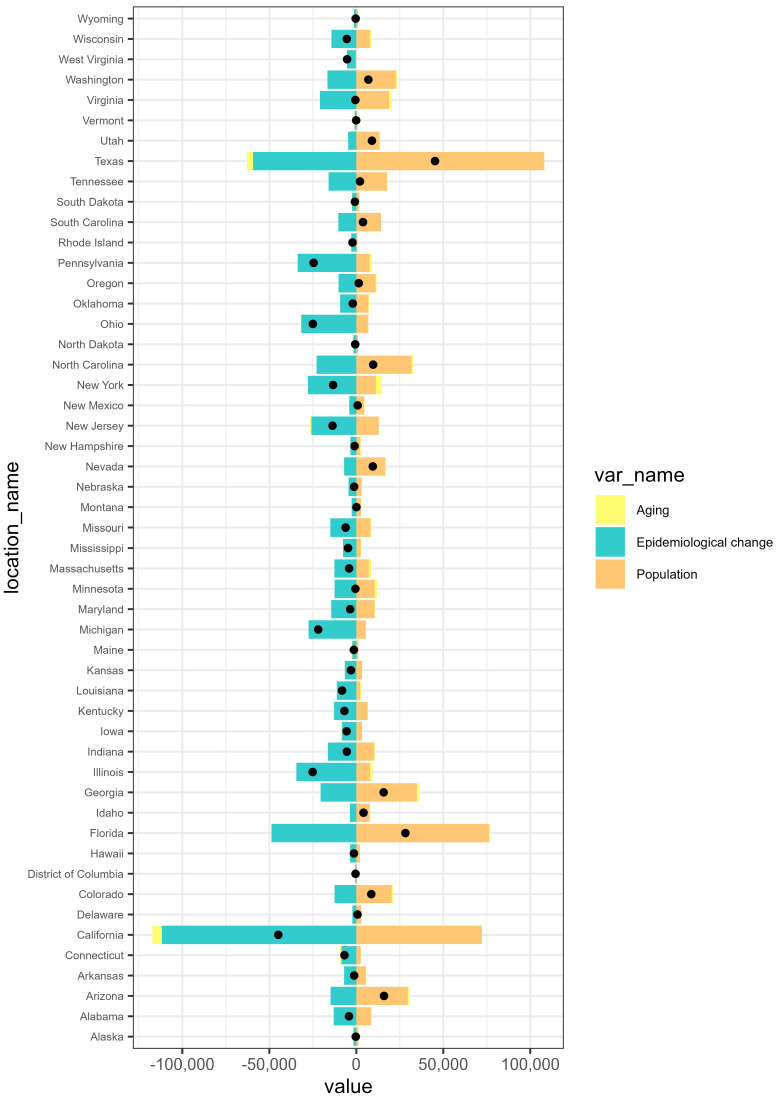
Decomposition analysis of IDID attributable to lead exposure for the 50 states and the District of Columbia.

Decomposition analyses of DALYs in the USA revealed that population growth led to an increase in the IDID burden attributable to lead exposure. In contrast, epidemiological and aging changes contributed to a reduction in DALYs. Among both males and females, DALYs attributable to lead exposure were negative, suggesting that the reductions due to epidemiological improvements and aging outweighed the increases from population growth (Figure 6). We also conducted a decomposition analysis for all 50 states and the District of Columbia. The effects of demography and epidemiology on DALYs showed variations across locations. The decomposition analysis indicates that in certain states, including California, New York, Illinois, Michigan, Pennsylvania, Massachusetts, and Missouri, epidemiological changes had the largest impact on this disease burden, contributing substantially to the overall reduction in DALYs. Conversely, population growth generally led to an increase in DALYs, as shown by Texas, Florida, Arizona, North Carolina, Georgia, Nevada, Washington, Oregon, and South Carolina. Aging had a relatively smaller impact in certain states, regardless of the direction of its effect on DALYs (Figure 7; Table S4).

### 3.5. Forecast of IDID Attributable to Lead Exposure in the USA till 2050

**Figure 8:**
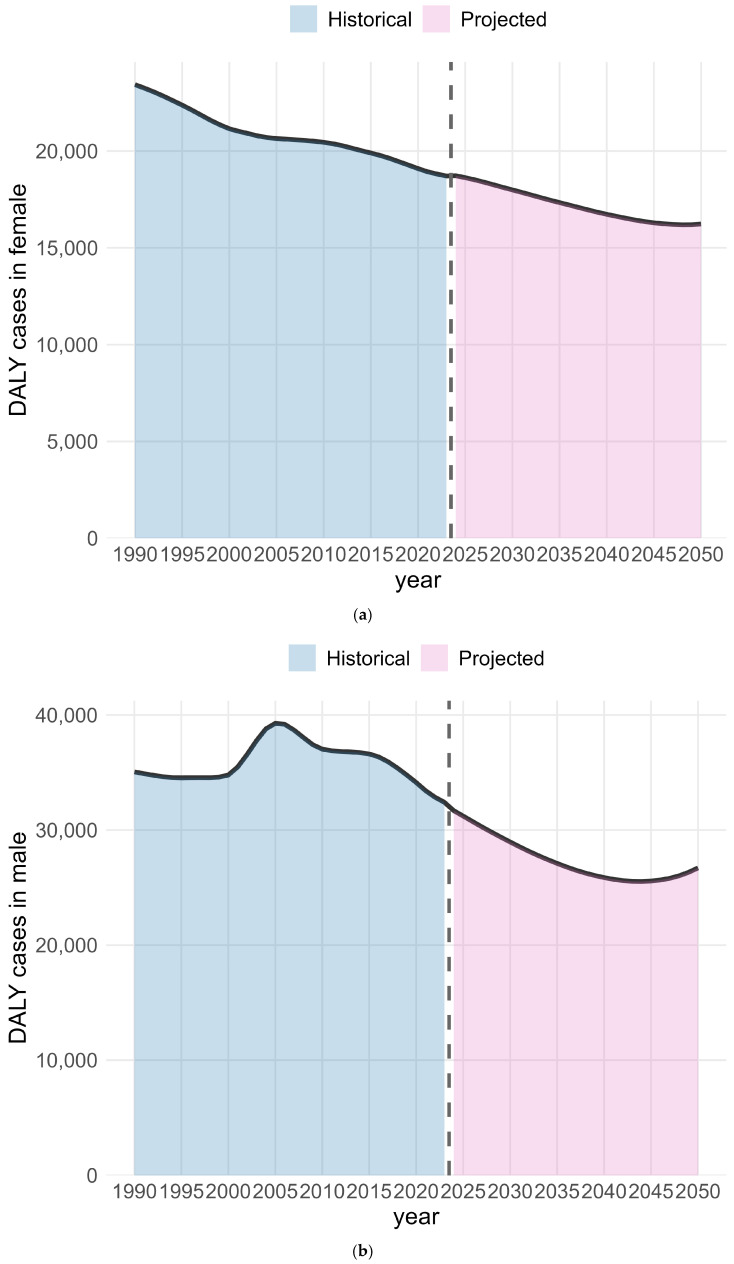

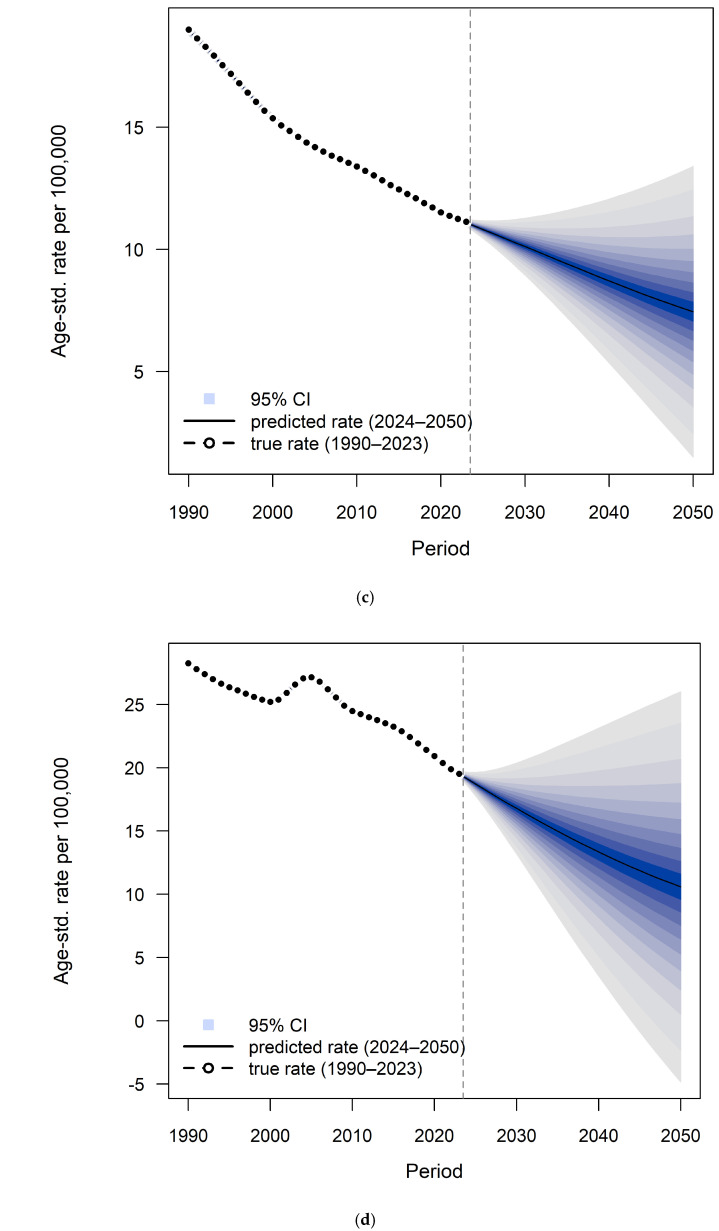
The DALYs and ASDR of the BAPC model of IDID attributable to lead exposure in the USA. (**a**) DALY cases in females; (**b**) DALY cases in males; (**c**) ASDR of females; (**d**) ASDR of males.

Based on the BAPC analysis, the DALYs (Figure 8a,b) and ASDR (Figure 8c,d) were predicted for this condition up to 2050 in the USA by sex (Table S5). Among females, DALYs declined continuously from 1990 to 2023 and are projected to continue decreasing until around 2048, with a slight increase thereafter through 2050. Among males, DALYs increased between 2000 and 2005 and then declined through 2023; they are projected to continue decreasing until approximately 2044, followed by a modest increase up to 2050. For females, the ASDR decreased from 1990 to 2023 and is projected to continue declining through 2050, while in males, the ASDR increased from 2000 to 2005 and declined during the remaining periods. It is expected that the ASDR will decrease to 7.45 for females and 10.59 for males per 100,000 population in 2050. ASDR is projected to continue declining for both sexes over the next 27 years. The reduction in DALYs and ASDR was greater among males than females from 1990 to 2023, with this pattern projected to extend to 2050. The backcasting validation was conducted for DALYs and ASDR in females and males by fitting the model to data from 1990 to 2018 and predicting values for 2019–2023. The resulting RMSE values were 84.53 for DALYs in females, 165.03 for DALYs in males, 0.11 for ASDR in females, and 0.12 for ASDR in males. The backcasting results based on the BAPC model for 2019–2023 are shown in Figures S52–S55. The observed and predicted values for 2019–2023 and the corresponding RMSEs are reported in Table S6.

## 4. Discussion

GBD modeling enables comparability across time and populations [36]. This study presents a systematic analysis of the epidemiological characteristics of IDID disease burden attributable to lead exposure in the USA. We estimated the DALYs and ASDR of lead exposure-induced IDID in the USA from 1990 to 2023, with projection up to 2050. From 1990 to 2023, on a national scale, the absolute number of DALYs and ASDR (AAPC < 0) has decreased for both sexes. Overall, the disease burden of IDID is higher in males than females from 1990 to 2023, as reflected by both the greater number of DALYs and higher ASDR. This finding corresponds to the global trend, which shows that males bore a higher disease burden of lead exposure-induced IDID [37]. Given that men have predominated in high-risk occupations for lead exposure, involving industry sectors, battery manufacturing, home renovations and so on, this occupational pattern likely contributes to the higher disease burden observed in males. The Occupational Safety and Health Administration (OSHA) has established standards and regulations for occupational lead exposure and these efforts have contributed to declines in adult blood lead levels (BLLs). Strengthening OSHA standards and ensuring stricter enforcement will be essential to further reduce occupational lead exposure and narrow these disparities [38,39].

Lead exposure is still a significant threat to public health on a global scale [38]. For developed countries, lead exposure occurs through environmental sources (78%), industrial sources (11%), and leaded paint (11%) [40]. To place U.S. trends in an international context, we compared ASDR across selected high-income countries using GBD 2023 estimates. The ASDR in Australia was 25.48 (95% UI: 10.14–48.45), while Switzerland, Japan, and Canada had lower rates of 12.81 (95% UI: 4.70–27.60), 6.26 (95% UI: 1.71–14.89), and 5.66 (95% UI: 1.63–13.40) respectively. A cohort study in New Zealand has reported an association between childhood lead exposure and lower cognitive ability and IQ loss, which is consistent with the findings of studies in the U.S. [12]. Canadian cohort studies revealed that BLLs were inversely associated with neurodevelopmental outcomes and intellectual function [41,42]. A GBD study from 1990 to 2019 reports that lead toxicity is prevalent in the U.S. [37]. Sources of lead contamination include gasoline and air, lead-based paint, toys and other consumer products, foods and diet. In addition, the workplace created a lead exposure hazard for workers, and take-home exposure is likely to elevate BLLs among US children younger than 6 years [43,44]. Lead exposure may also begin prenatally, as maternal contact with contaminated sources can result in lead crossing the placental barrier, exerting toxicity on the fetus [45].

In our study, by age group, we observed a declining disease burden among children and adolescents, whereas older adults experienced an increase over the study period. The rising burden in older adults is consistent with the findings of He et al., who reported a gradual shift in disease burden from children toward older populations since 1990 [26]. This results suggest that lead stored in bone continues to be mobilized for decades after external exposures have declined [46]. Legacy exposure to lead, especially among workers with long occupational histories, is likely to keep causing adverse health effects, even though present-day exposure levels are reduced [47]. Lead exposure during childhood is associated with persistent decreased cognitive function. Administrative data from 1940 indicated that U.S. adults who lived in lead-exposed cities during childhood experienced poorer cognitive function later in life [48]. In contrast to the pattern observed in older adults, we also noted, based on GBD estimates, that the childhood IDID burden is lower in the United States than in China [49]. This is likely attributable to the early implementation of lead control measures in the United States in the 1970s, including the early phaseout of leaded gasoline, the early ban on residential lead-based paint and subsequent regulatory measures [50,51]. More recently, enhancements in blood lead surveillance have strengthened the early identification of at-risk children. The CDC lowered the blood lead reference value (BLRV) in children from 5.0 to 3.5 μg/dL in 2021, underscoring ongoing efforts to promote early detection and prevention [52]. In addition, providing blood lead screening tests and secondary prevention are effective ways for children to avoid IQ loss and other harmful effects [53,54]. A study by Delgado et al. has indicated the association between lead exposure and a broad range of intellectual outcomes and educational attainment and further suggests that elevated blood lead levels increase the likelihood of meeting diagnostic criteria for intellectual disabilities in early childhood [55]. Prospective cohort studies using individual-level data have consistently demonstrated a causal association between lead exposure and impaired intellectual development in U.S. children [10,56]. The burden of lead exposure-induced IDID varies across different states and regions. Data shows that California remained the state with the highest DALYs for both 1990 and 2023. In California, lead contamination has a serious impact on households with low socioeconomic status as well as households of color and of immigrants, such as Hispanic residents. In socioeconomically disadvantaged communities, lead disproportionately affects the poorer populations, creating problems that are often overlooked by policymakers. Additionally, historic use of lead gasoline explained the reason for elevated lead concentrations near Californian highways [57,58]. Redlining, discriminatory land use and housing inequities, as well as disproportionate exposure to pollution sources in low-income communities, have contributed to racial and ethnic disparities in lead exposure over time [59]. A nationwide assessment of environmental justice trends in public school students revealed inequities with lead exposure, and these may be attributable to variance in local histories of immigration, housing segregation, land-use policies, urban development, and industrial activity [60]. Applying an environmental justice framework allows these disparities to be examined in a broader context, extending beyond childhood lead poisoning [61].

A study by Armatas et al. reported that occupational exposure has a serious impact on Californian workers [62]. The ASDR demonstrated spatial heterogeneity, with Arkansas recording the highest values for both 1990 and 2023. This pattern may in part result from Arkansas being the only state without an implied warranty of habitability in landlord–tenant law, a policy gap that can contribute to substandard rental housing and residential conditions that are more conducive to lead exposure and its associated health risks [63]. A high ASDR was also observed in the Midwestern “lead belt,” spanning southwest Missouri and parts of northeast Oklahoma, linked to a long history of lead contamination from mining and smelting activities [64].

A recent study by Hauptman et al. has demonstrated a graded association between neighborhood poverty and housing built before the 1950s (prior to the ban on lead-based paint in 1978) and increased odds of lead poisoning in the U.S. [65]. Elevated BLLs among black children may be explained by increased exposure to lead-contaminated household dust and substandard housing conditions, including deteriorating interior paint and flooring [66]. Research findings from North Carolina suggest that children from socio-economically disadvantaged households experience systematically higher lead exposure. This disadvantage, reflected in their enrollment in the free/reduced price lunch program at school and lower parental education, may be associated with poorer academic performance in elementary school [67]. Exposure pathways appear to differ between urban and rural settings. Road coverage contributed to higher BLLs in urban areas but not in rural areas. Urban children are more likely to be exposed through lead from house dust and legacy lead in the outdoor environment, while dietary intake and drinking water is an identified source for rural children [68]. Urban expansion, together with urban heat island effects, has been associated with a rising risk of lead poisoning [69]. Racial patterns also differed across contexts, with higher blood lead levels among black children in the urban areas and among white children in the rural areas [68]. Black and Hispanic/Latino populations experience higher rates of lead poisoning, largely associated with unemployment and older housing conditions [69]. Evidence in Cleveland, Ohio, suggests that housing-based lead mitigation interventions are cost-effective because the long-term social and economic costs of childhood lead exposure, including reduced lifetime earnings and increased risks of neuropsychiatric and educational impairments, substantially exceed the costs of prevention, particularly in communities affected by structural racism [70]. One study indicated that parents’ health literacy serves as a potential barrier to lead poisoning prevention [71]. Residents without authorized legal status may face uncertainty regarding their rights to assess and address the lead hazards in their homes [72]. An APC model was used to analyze the ASDR for this condition in the U.S. population from 1990 to 2023. The age effect exhibited a peak at approximately 20 years of age and then a gradual decrease with advancing age. The cohort effect showed positive deviations among cohorts born between the 1910s and 1960s, a period characterized by limited prenatal screening and restricted access to specialized fetal healthcare [73,74]. Additionally, the decreasing trend with later birth cohorts likely suggests the effective progression of standards and regulations that contributed to the reduction in environmental lead exposure in more recent years. A gradual decline in the period effect was also observed in recent years, suggesting progress in preventive public health efforts.

Decomposition analyses revealed that population growth contributed to an increase in DALYs of IDID attributable to lead exposure in both sexes at the national level in the United States, but this effect is less pronounced than the decreases associated with epidemiological and demographic changes. This may be the results of effective implementation of environmental policies and minimization of lead use. This pattern is consistent with states such as California and New York, where epidemiological improvements may have contributed to a reduction in the disease burden. Conversely, in states experiencing rapid population growth and a substantial foreign-born population, such as Texas and Florida, the contribution of population expansion offset much of the progress achieved through epidemiological improvements. This suggests that health gains from environmental and policy interventions may be undermined by demographic pressure. Moreover, population growth in these regions often results from the influx of young families and migrants, groups that may experience higher socioeconomic vulnerability, including substandard housing conditions and increased childhood lead poisoning risks [75,76]. Overall, the spatial disparities emphasize the need for public health strategies that integrate demographic trends and socioeconomic inequalities.

The BAPC analysis indicates that IDID attributable to lead exposure in the USA is anticipated to show a sustained downward trend from 2024 to 2050, reflected by reductions in the ASDR for both females and males. Males are projected to experience a more pronounced decline, as shown by the larger reductions in DALYs and ASDR. The steeper projected declines among males may reflect their higher historical exposure, particularly in environments with greater contact with lead-contaminated dust, soil, and aging housing. A higher baseline burden creates more room for reduction as legacy hazards continue to be addressed. The smaller decrease anticipated for females is consistent with their generally lower exposure levels. Physiological factors may also contribute. Prior research has shown that during periods of accelerated bone turnover, especially the menopausal transition, lead stored in bone can be remobilized into the bloodstream [77]. The validity of the BAPC forecasting model was supported by the backcasting analysis. The predicted values were close to the observed data, as reflected by the relatively small RMSE. This suggests that the BAPC model is suitable for describing recent trends in our data.

From 1976–1980 to 2015–2016, the geometric mean blood lead level (BLL) of the US population dropped by approximately 93.6%. This substantial reduction underscores the effectiveness of evidence-based public health practices and regulatory actions aimed at minimizing lead exposure [38]. In line with this evidence, our study indicated the role of past regulatory interventions in decreasing the disease burden of IDID attributable to lead exposure in the USA. Public health strategies should be further strengthened to address residual risks. Given the tendency for lead release from the skeleton in female adults, especially at their postmenopausal phase, calcium supplementation may help mitigate lead release from bone. In addition, findings from a cohort study confirmed that skeletal lead is released at an increased rate during pregnancy and can transfer to the fetus [78]. A randomized placebo-controlled trial reported that calcium supplementation was associated with modest reductions in blood lead during pregnancy and may represent a secondary prevention measure to reducing maternal circulating lead and subsequent fetal exposure [79]. Based on these findings, we recommend that public health strategies should prioritize reducing the intensity of bone resorption and investigating key determinants, such as ethnicity, occupation, residential environment, education, lifestyle behaviors, and water quality, that influence bone and blood lead levels in women and may exacerbate the effects of postmenopausal osteoporosis [80]. In addition, meta-analysis suggests a causal association between blood lead levels and iron deficiency anemia (IDA). Lead preferentially binds to red blood cells, leading to its accumulation in the hematopoietic system. Children with IDA may be at increased risk of subsequent lead poisoning [81]. Greater screening efforts should be directed to at-risk populations, especially those with socioeconomic vulnerabilities. Universal testing for blood lead levels is recommended. Federal policy also mandates age-specific testing for young children enrolled in Medicaid [10,11].

This study provides a comprehensive analysis of the disease burden of lead exposure-related IDID in the United States of America based on the most up-to-date data. The age-, sex-, and region-specific results offer insights for public health by providing guidance on optimizing resource allocation and informing evidence-based decision making. However, it is not free from limitations. In the GBD framework, lead exposure is defined as the risk factor, whereas IDID is classified as a disease category in the GBD cause hierarchy. Under this structure, the GBD database does not provide estimates for other epidemiologic measures such as prevalence, incidence, and mortality. In addition, since DALYs consist entirely of YLDs for this condition, the study scope and measurement were limited. DALYs and the ASDR capture overall population burden effectively, but they do not represent individual-level risk; therefore, the measures may overlook disparities among marginalized populations, such as racial/ethnic minorities, immigrants and refugees [82]. Although studies have consistently shown that lead exposure is associated with impaired intellectual development in children [10,55,56], our analysis relies on population-level estimates derived from the GBD framework and therefore reflects modeled risk attribution rather than definitive causal effects. Because this study relies exclusively on GBD modeled data, the burden estimates remain dependent on assumptions underlying the exposure–outcome relationships and entail inherent uncertainties in data input [13,14,83]. The inability to conduct additional sensitivity analyses or to explicitly adjust for key potential confounders, including socioeconomic status, healthcare access, nutritional factors, and genetic susceptibility, means that results may be influenced by unmeasured co-exposure and individual susceptibilities [84,85]. Wider uncertainty intervals are common in regions with limited data, indicating a lower level of predictive confidence and increased variability in the information. Furthermore, because the outcomes presented in this paper are related to the U.S., specific recommendations and strategies will be tailored to smaller geographical areas and will depend on local policy contexts and budgetary constraints. Finally, our projections to 2050 using the BAPC model are based on historical trends and may not account for future changes in policy or exposure pathways and therefore may not provide a fully accurate estimate of the future burden.

## 5. Conclusions

In summary, the study provides a comprehensive analysis of the USA’s IDID burden attributable to lead exposure. There was an overall decrease in the national disease burden, reflected by a decline in ASDR during 1990–2023, and continued decreases were projected through 2050 for both females and males. Males bore a higher burden, largely attributable to the greater likelihood of occupational exposure to lead. Over the study period, females showed a slower decline in disease burden than that of males, which may result from their bone resorption due to age-related hormones. Public health authorities need to consolidate the effect of the control of occupational exposure. Greater efforts should be made, including reducing the intensity of bone resorption, identifying modifying factors, and providing nutritional supplements. Implementation of these strategies will facilitate the reduction in this disease burden in vulnerable populations.

## Figures and Tables

**Table 1:** DALY number and ASDR of IDID attributable to lead exposure between 1990 and 2023 by gender and states.

Characteristics		DALYs			ASDR per 100,000	
United States of America	Number in 1990 (95% UI)	Number in 2023 (95% UI)	Percentage change (%)	ASDR in 1990 (95% UI)	ASDR in 2023 (95% UI)	Percentage change (%)
Sex	58,508 (21,112; 122,048)	51,194 (19,265; 98,846)	−12.50%	24.21 (8.8; 50.25)	15.14 (5.89; 31.5)	−37.46%
Female	23,435 (9264; 46,235)	18,750 (7437; 36,177)	−19.99%	19.45 (7.68; 38.15)	10.97 (4.47; 22.7)	−43.60%
Male	35,073 (12,018; 74,896)	32,444 (11,827; 64,166)	−7.50%	28.91 (9.9; 61.5)	19.26 (7.31; 40.29)	−33.38%

## Data Availability

All data analyzed in this study is shared publicly in the GBD Results Tool (http://ghdx.healthdata.org/gbd-results-tool, accessed on 6 November 2025).

## Supplementary Materials

The following supporting information can be downloaded at: https://www.mdpi.com/article/10.3390/healthcare14040508/s1, Figure S1. Joinpoint analysis of IDID attributable to lead exposure in Alabama from 1990 to 2023; Figure S2. Joinpoint analysis of IDID attributable to lead exposure in Alaska from 1990 to 2023; Figure S3. Joinpoint analysis of IDID attributable to lead exposure in Arizona from 1990 to 2023; Figure S4. Joinpoint analysis of IDID attributable to lead exposure in Arkansas from 1990 to 2023; Figure S5. Joinpoint analysis of IDID attributable to lead exposure in California from 1990 to 2023; Figure S6. Joinpoint analysis of IDID attributable to lead exposure in Colorado from 1990 to 2023; Figure S7. Joinpoint analysis of IDID attributable to lead exposure in Connecticut from 1990 to 2023; Figure S8. Joinpoint analysis of IDID attributable to lead exposure in Delaware from 1990 to 2023; Figure S9. Joinpoint analysis of IDID attributable to lead exposure in the District of Columbia from 1990 to 2023; Figure S10. Joinpoint analysis of IDID attributable to lead exposure in Florida from 1990 to 2023; Figure S11. Joinpoint analysis of IDID attributable to lead exposure in Georgia from 1990 to 2023; Figure S12. Joinpoint analysis of IDID attributable to lead exposure in Hawaii from 1990 to 2023; Figure S13. Joinpoint analysis of IDID attributable to lead exposure in Idaho from 1990 to 2023; Figure S14. Joinpoint analysis of IDID attributable to lead exposure in Illinois from 1990 to 2023; Figure S15. Joinpoint analysis of IDID attributable to lead exposure in Indiana from 1990 to 2023; Figure S16. Joinpoint analysis of IDID attributable to lead exposure in Iowa from 1990 to 2023; Figure S17. Joinpoint analysis of IDID attributable to lead exposure in Kansas from 1990 to 2023; Figure S18. Joinpoint analysis of IDID attributable to lead exposure in Kentucky from 1990 to 2023; Figure S19. Joinpoint analysis of IDID attributable to lead exposure in Louisiana from 1990 to 2023; Figure S20. Joinpoint analysis of IDID attributable to lead exposure in Maine from 1990 to 2023; Figure S21. Joinpoint analysis of IDID attributable to lead exposure in Maryland from 1990 to 2023; Figure S22. Joinpoint analysis of IDID attributable to lead exposure in Massachusetts from 1990 to 2023; Figure S23. Joinpoint analysis of IDID attributable to lead exposure in Michigan from 1990 to 2023; Figure S24. Joinpoint analysis of IDID attributable to lead exposure in Minnesota from 1990 to 2023; Figure S25. Joinpoint analysis of IDID attributable to lead exposure in Mississippi from 1990 to 2023; Figure S26. Joinpoint analysis of IDID attributable to lead exposure in Missouri from 1990 to 2023; Figure S27. Joinpoint analysis of IDID attributable to lead exposure in Montana from 1990 to 2023; Figure S28. Joinpoint analysis of IDID attributable to lead exposure in Nebraska from 1990 to 2023; Figure S29. Joinpoint analysis of IDID attributable to lead exposure in Navada from 1990 to 2023; Figure S30. Joinpoint analysis of IDID attributable to lead exposure in New Hampshire from 1990 to 2023; Figure S31. Joinpoint analysis of IDID attributable to lead exposure in New Jersey from 1990 to 2023; Figure S32. Joinpoint analysis of IDID attributable to lead exposure in New Mexico from 1990 to 2023; Figure S33. Joinpoint analysis of IDID attributable to lead exposure in New York from 1990 to 2023; Figure S34. Joinpoint analysis of IDID attributable to lead exposure in North Carolina from 1990 to 2023; Figure S35. Joinpoint analysis of IDID attributable to lead exposure in New Dakota from 1990 to 2023; Figure S36. Joinpoint analysis of IDID attributable to lead exposure in Ohio from 1990 to 2023; Figure S37. Joinpoint analysis of IDID attributable to lead exposure in Oklahoma from 1990 to 2023; Figure S38. Joinpoint analysis of IDID attributable to lead exposure in Oregon from 1990 to 2023; Figure S39. Joinpoint analysis of IDID attributable to lead exposure in Pennsylvania from 1990 to 2023; Figure S40. Joinpoint analysis of IDID attributable to lead exposure in Rhode Island from 1990 to 2023; Figure S41. Joinpoint analysis of IDID attributable to lead exposure in South Carolina from 1990 to 2023; Figure S42. Joinpoint analysis of IDID attributable to lead exposure in South Dakota from 1990 to 2023; Figure S43. Joinpoint analysis of IDID attributable to lead exposure in Tennessee from 1990 to 2023; Figure S44. Joinpoint analysis of IDID attributable to lead exposure in Texas from 1990 to 2023; Figure S45. Joinpoint analysis of IDID attributable to lead exposure in Utah from 1990 to 2023; Figure S46. Joinpoint analysis of IDID attributable to lead exposure in Vermont from 1990 to 2023; Figure S47. Joinpoint analysis of IDID attributable to lead exposure in Virginia from 1990 to 2023; Figure S48. Joinpoint analysis of IDID attributable to lead exposure in Washington from 1990 to 2023; Figure S49. Joinpoint analysis of IDID attributable to lead exposure in West Virginia from 1990 to 2023; Figure S50. Joinpoint analysis of IDID attributable to lead exposure in Wisconsin from 1990 to 2023; Figure S51. Joinpoint analysis of IDID attributable to lead exposure in Wyoming from 1990 to 2023; Figure S52. Observed and predicted DALYs in females from the backcasting analysis (2019–2023); Figure S53. Observed and predicted DALYs in males from the backcasting analysis (2019–2023); Figure S54. Observed and predicted ASDR in females from the backcasting analysis (2019–2023); Figure S55. Observed and predicted ASDR in males from the backcasting analysis (2019–2023). Table S1: State-level IDID attributable to lead exposure from 1990 to 2023 and AAPC for IDID attributable to lead exposure in the USA from 1990 to 2023; Table S2: Age-specific DALY numbers and rates (per 100,000) for IDID attributable to lead exposure in the USA in 1990 and 2023; Table S3: APC analysis for IDID attributable to lead exposure in the USA from 1990 to 2023; Table S4: Detailed results of the decomposition analysis for IDID attributable to lead exposure in the USA from 1990 to 2023; Table S5: Projected DALYs and ASDR attributable to lead exposure-induced IDID in the USA, 2024–2050, by sex. Table S6: BAPC analyses for backcasting validations and RMSE.

## Abbreviations

The following abbreviations are used in this manuscript:

GBD	Global Burden of Disease
ID	Intellectual Disability
IDID	Idiopathic Developmental Intellectual Disability
IHME	Institute for Health Metrics and Evaluation
IQ	Intelligence Quotient
DALY	Disability-Adjusted Life Year
ASDR	Age-Standardized DALY Rate
YLD	Year Lived With Disability
BLL	Blood Lead Level
YLL	Year Of Life Lost
AAPC	Average Annual Percentage Change
UIs	Uncertainty Intervals
CI	Confidence Interval
APC	Age–Period–Cohort
BAPC	Bayesian Age–Period–Cohort
INLA	Integrated Nested Laplace Approximation
OSHA	Occupational Safety and Health Administration
CDC	Centers for Disease Control and Prevention
RMSE	Root Mean Squared Error
PAFs	Population Attributable Fractions
IDA	Iron deficiency anemia
