# Supplementary material for: The Burdens of Idiopathic Developmental Intellectual Disability Attributable to Lead Exposure from 1990 to 2023 and a Projection to 2050 in the USA: Findings from the Global Burden of Disease Study 2023
Source: Healthcare (Basel). 2026 Feb 16;14(4):508. doi: 10.3390/healthcare14040508 (PMC12940874; doi:10.3390/healthcare14040508)
Supplement: Supplementary file 1 [file healthcare-14-00508-s001.zip › Supplementary Figure Legends.pdf]

Supplementary Figures

The Burdens of Idiopathic Developmental Intellectual Disability Attributable to Lead Exposure from 1990 to 2023 and Projection to 2050 in the USA: Findings from the Global Burden of Disease Study 2023

Supplementary figure legends

Figure S1. Joinpoint analysis of IDID attributable to Pb exposure in Alabama from 1990 to 2023

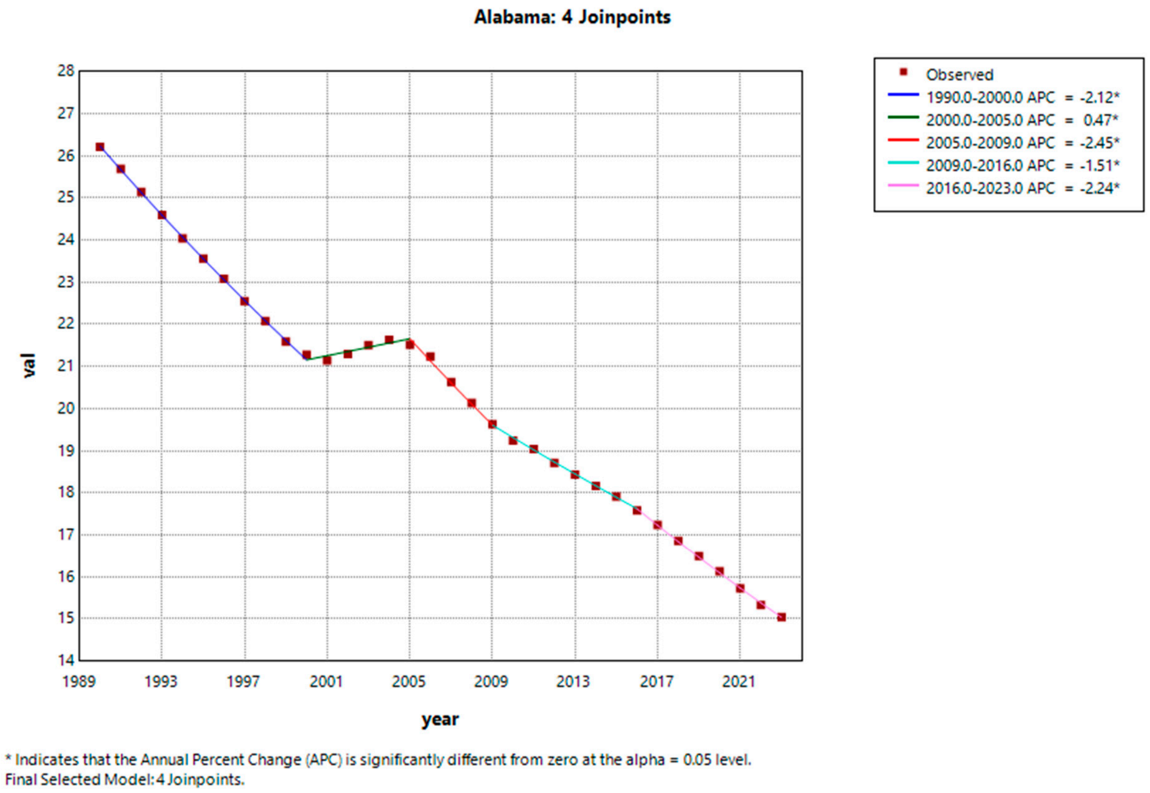

Figure S2. Joinpoint analysis of IDID attributable to Pb exposure in Alaksa from 1990 to 2023

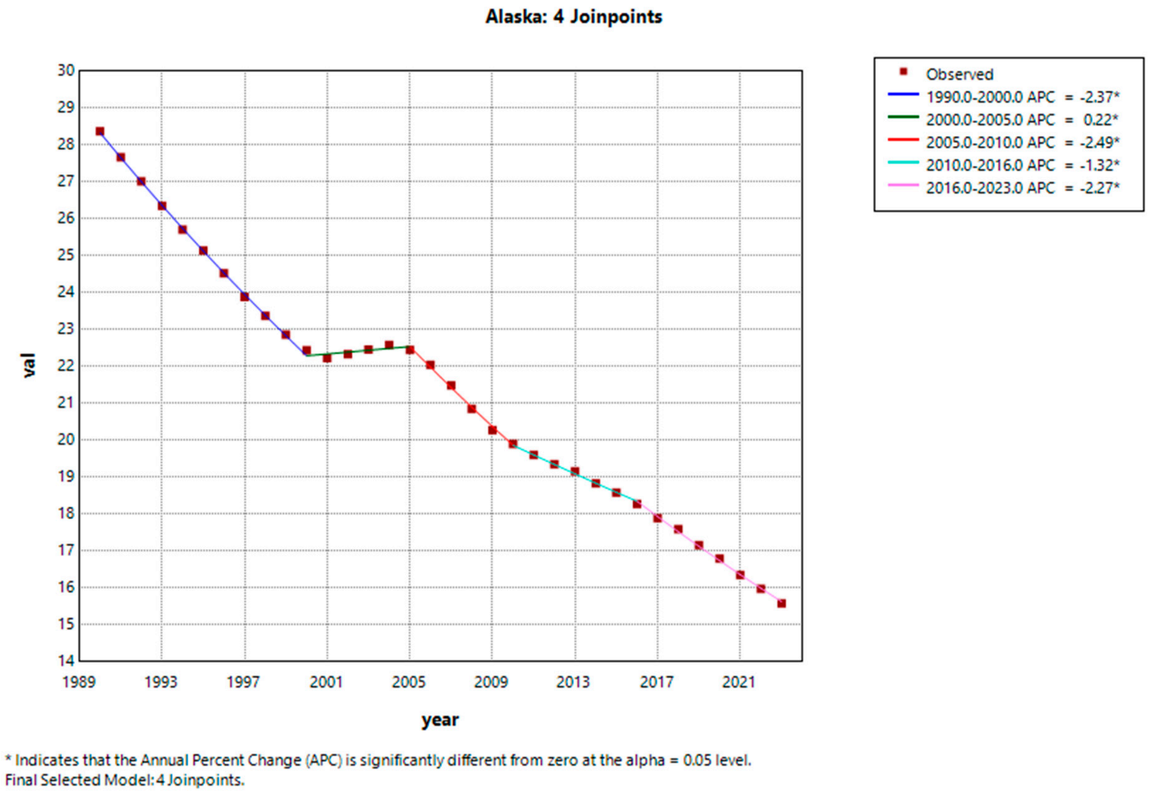

Figure S3. Joinpoint analysis of IDID attributable to Pb exposure in Arizona from 1990 to 2023

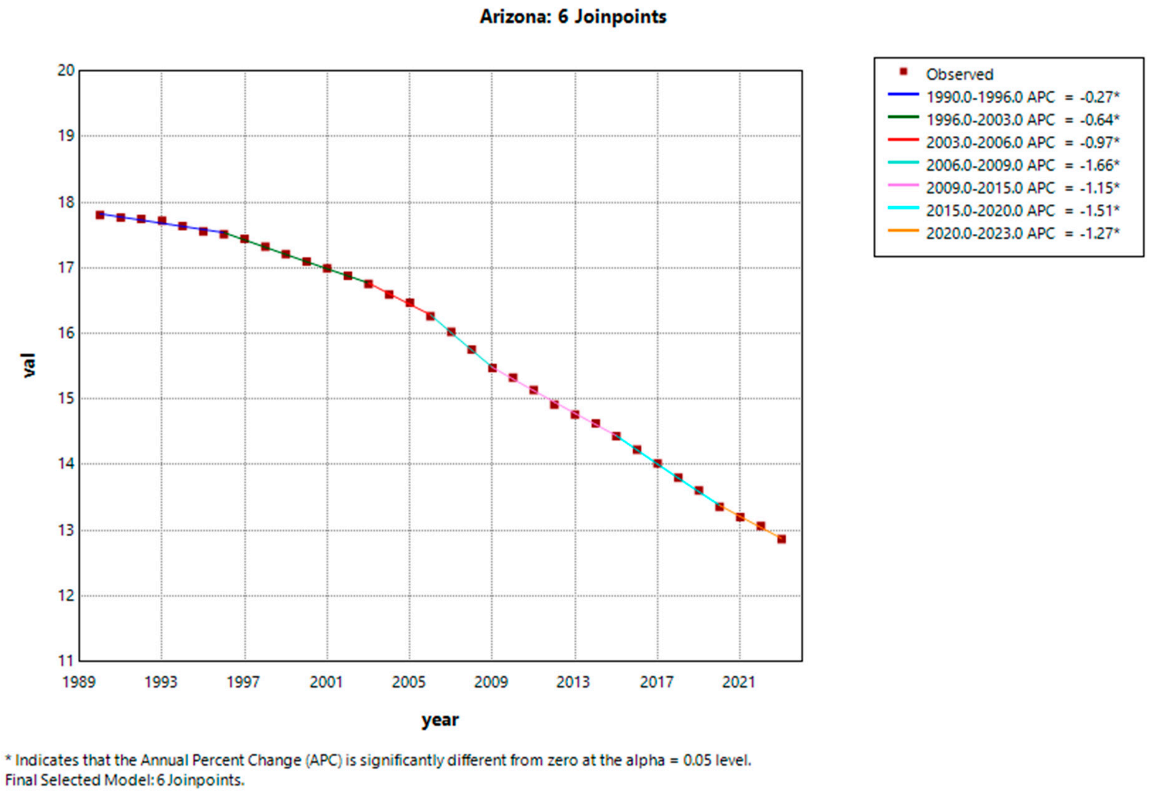

Figure S4. Joinpoint analysis of IDID attributable to Pb exposure in Arkansas from 1990 to 2023

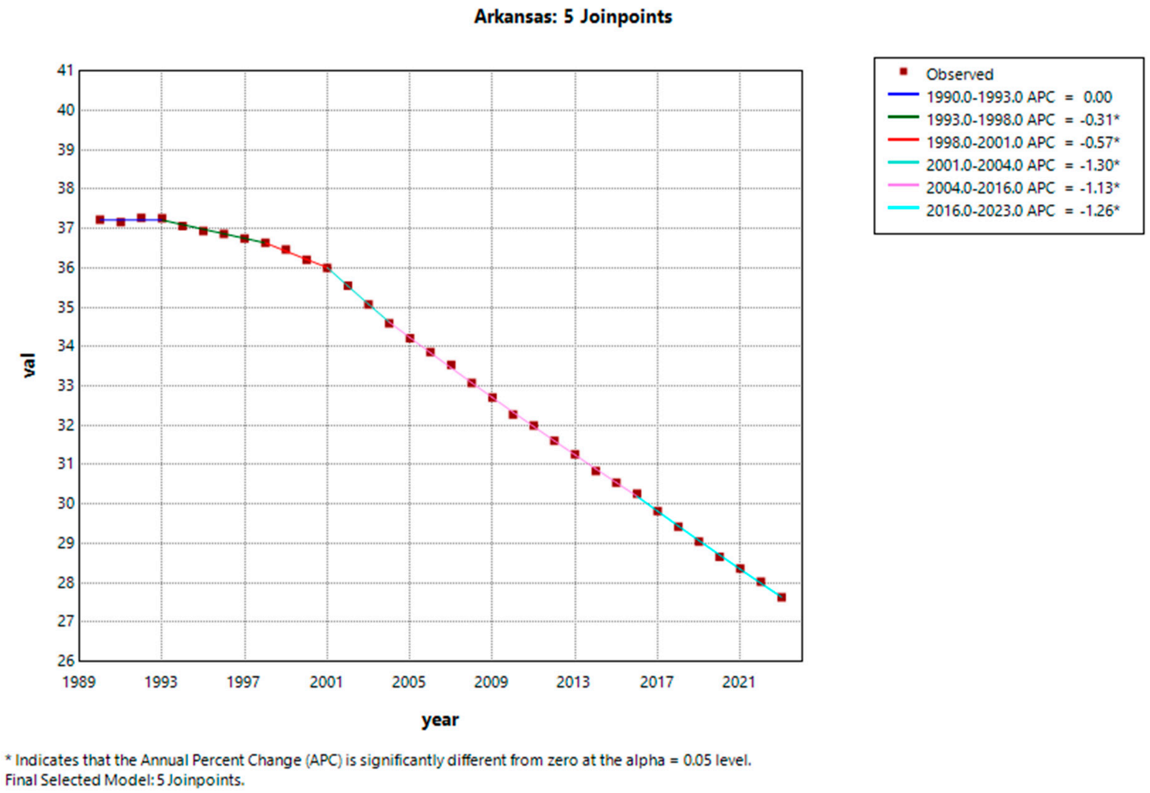

Figure S5. Joinpoint analysis of IDID attributable to Pb exposure in California from 1990 to 2023

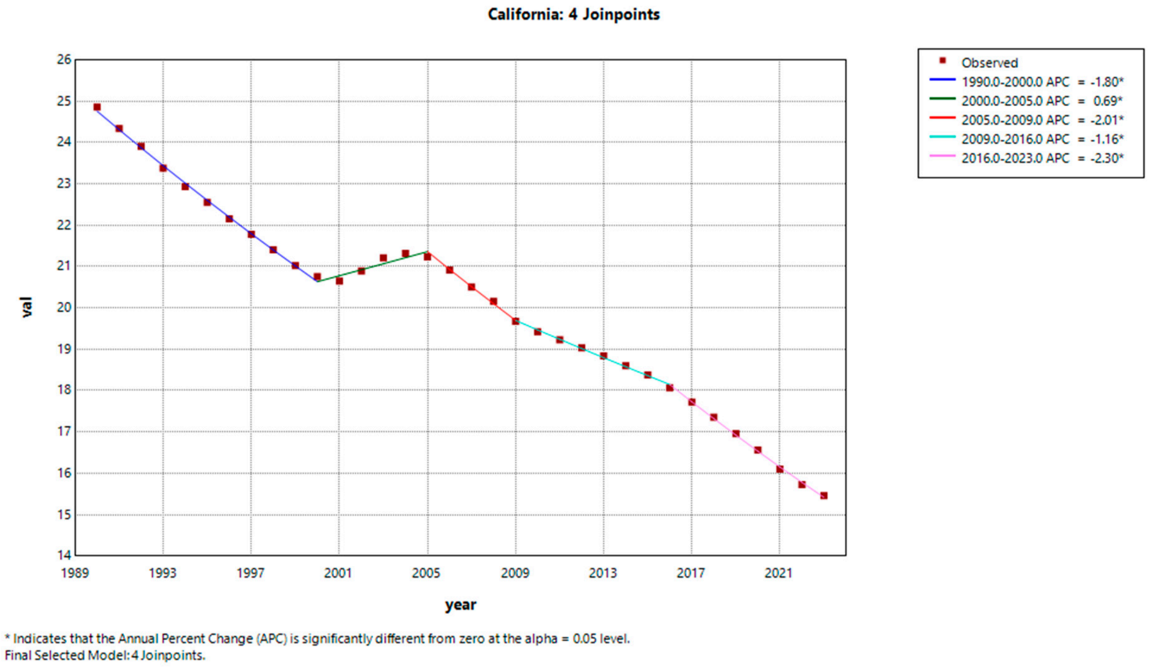

Figure S6. Joinpoint analysis of IDID attributable to Pb exposure in Colorado from 1990 to 2023

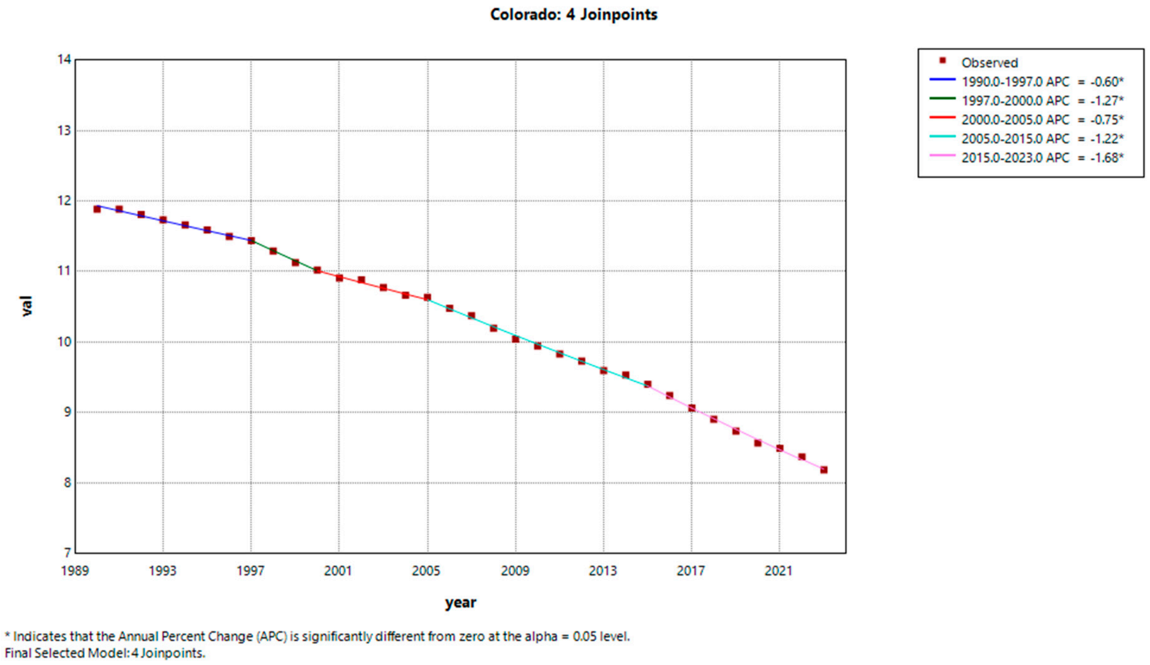

Figure S7. Joinpoint analysis of IDID attributable to Pb exposure in Connecticut from 1990 to 2023

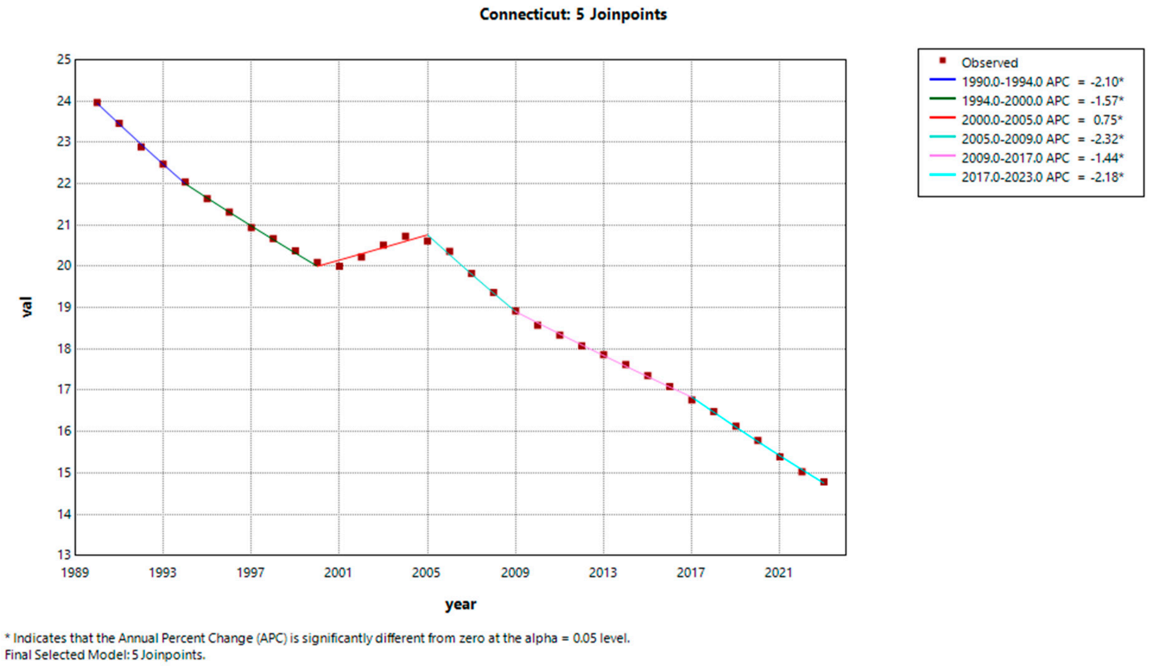

Figure S8. Joinpoint analysis of IDID attributable to Pb exposure in Delaware from 1990 to 2023

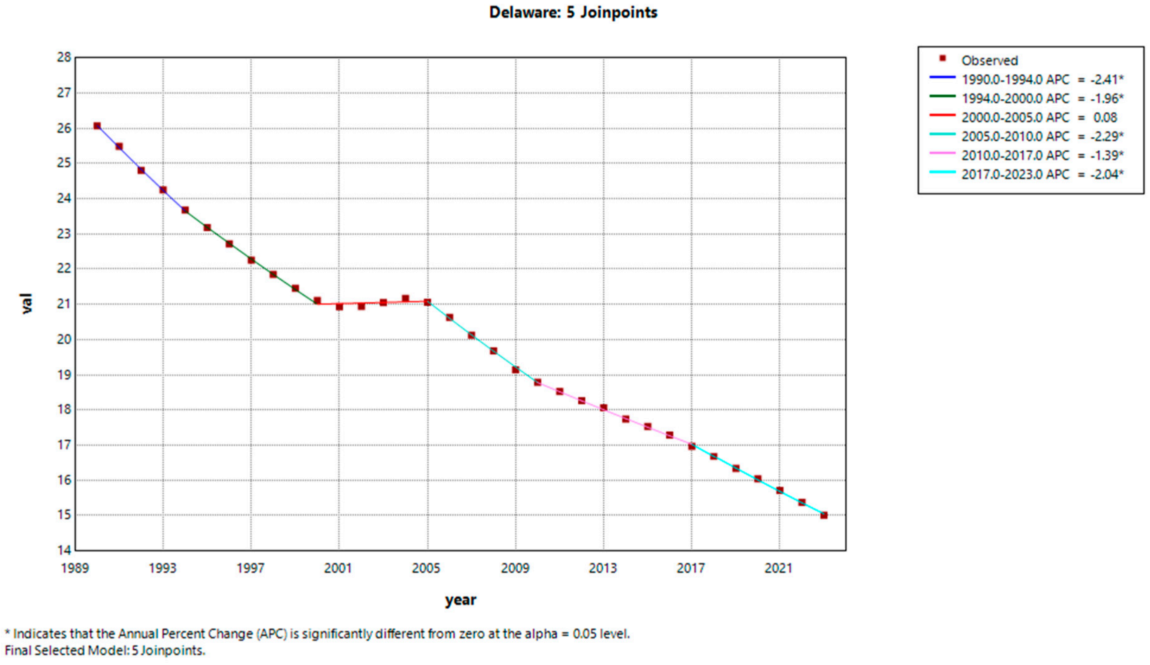

Figure S9. Joinpoint analysis of IDID attributable to Pb exposure in District of Columbia from 1990 to 2023

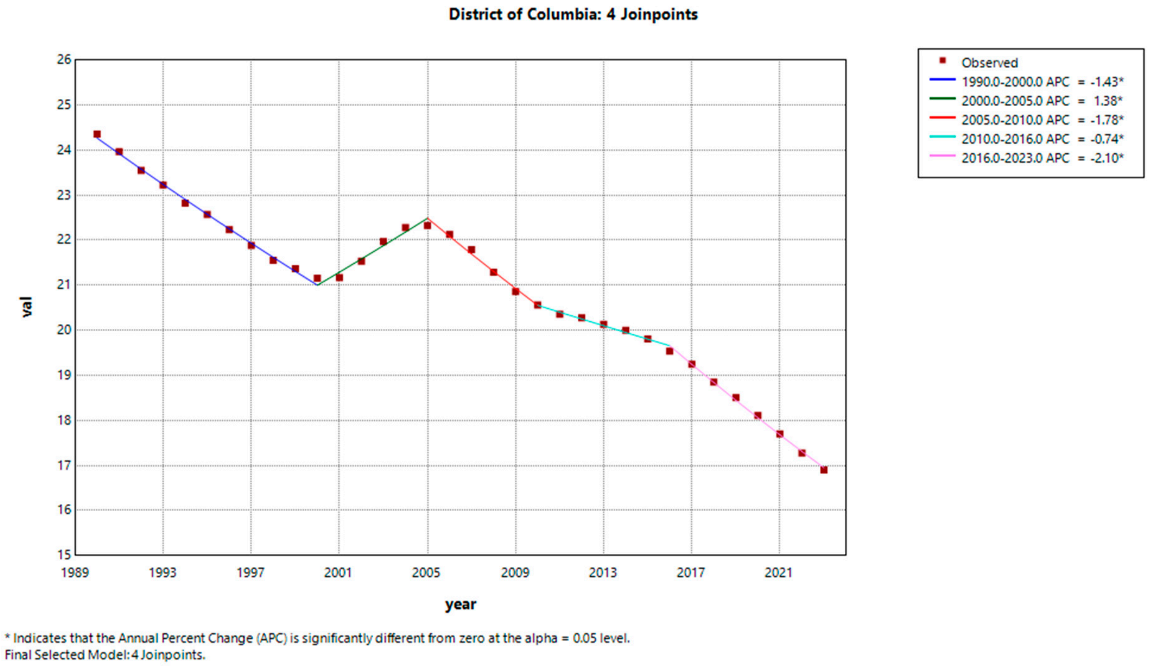

Figure S10. Joinpoint analysis of IDID attributable to Pb exposure in Florida from 1990 to 2023

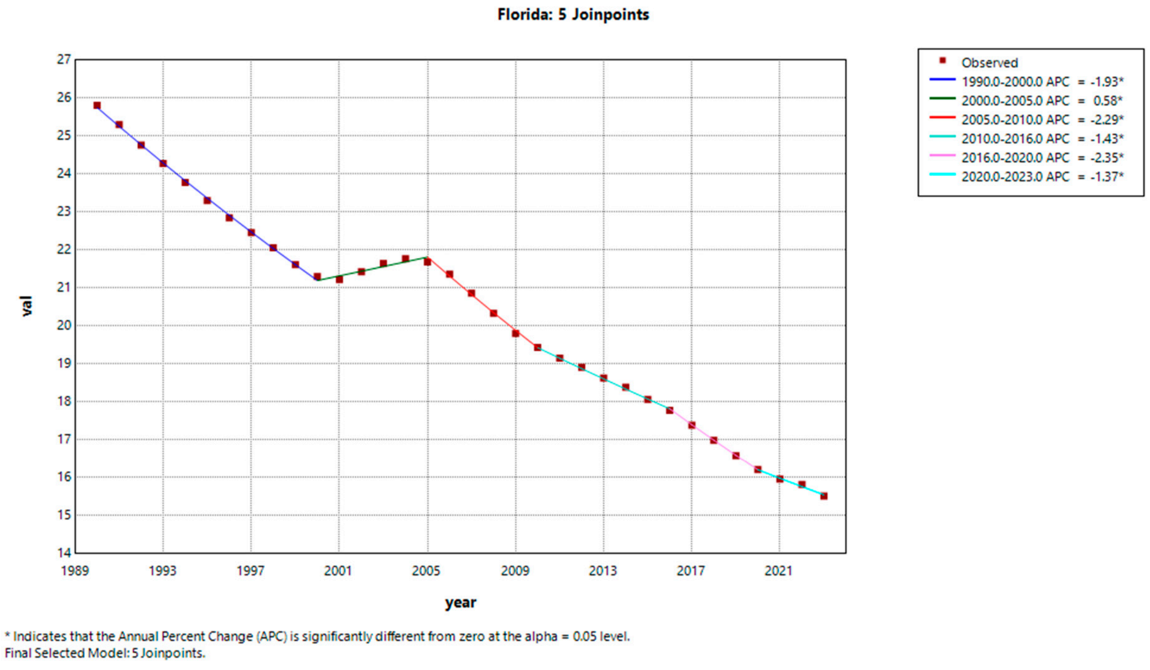

Figure S11. Joinpoint analysis of IDID attributable to Pb exposure in Georgia from 1990 to 2023

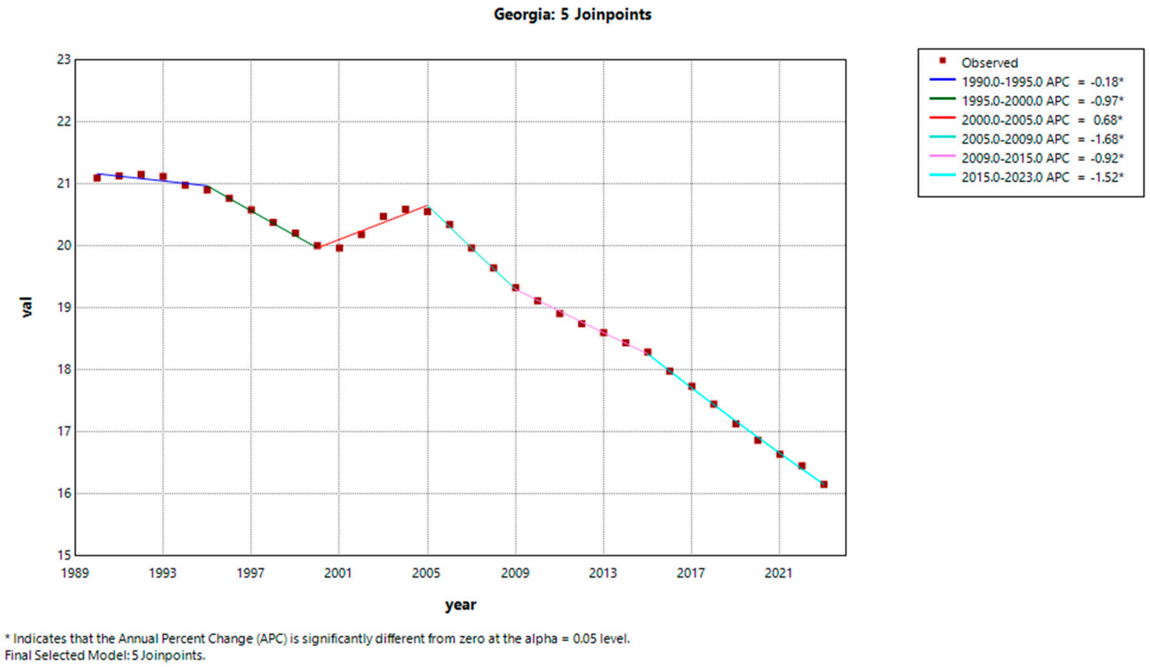

Figure S12. Joinpoint analysis of IDID attributable to Pb exposure in Hawaii from 1990 to 2023

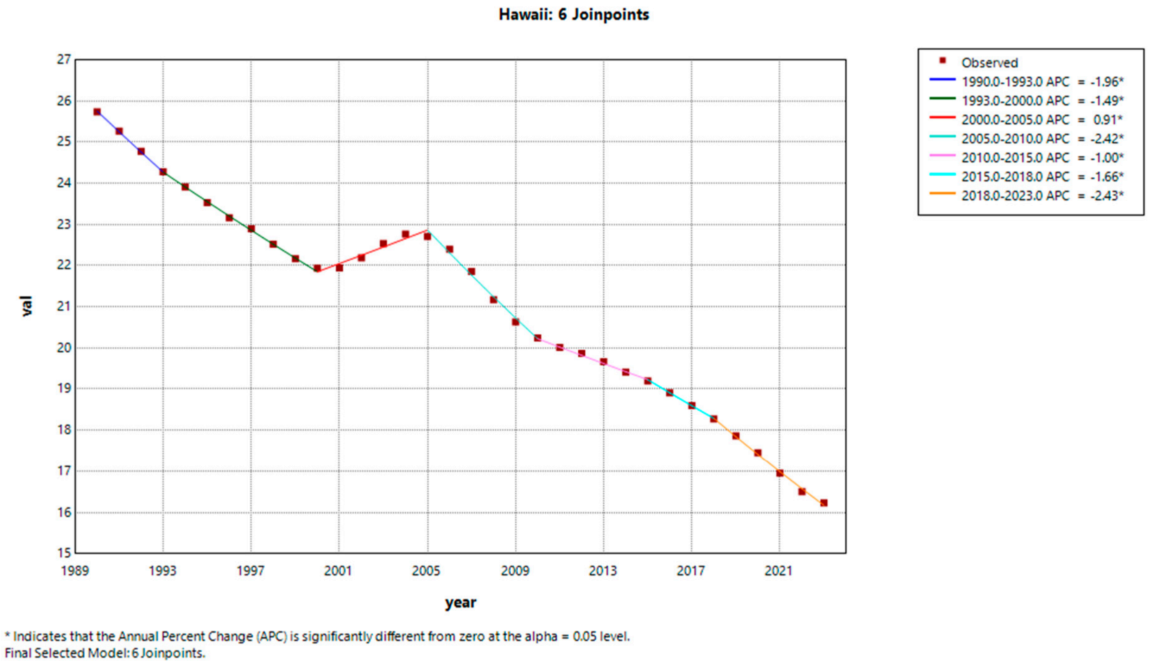

Figure S13. Joinpoint analysis of IDID attributable to Pb exposure in Idaho from 1990 to 2023

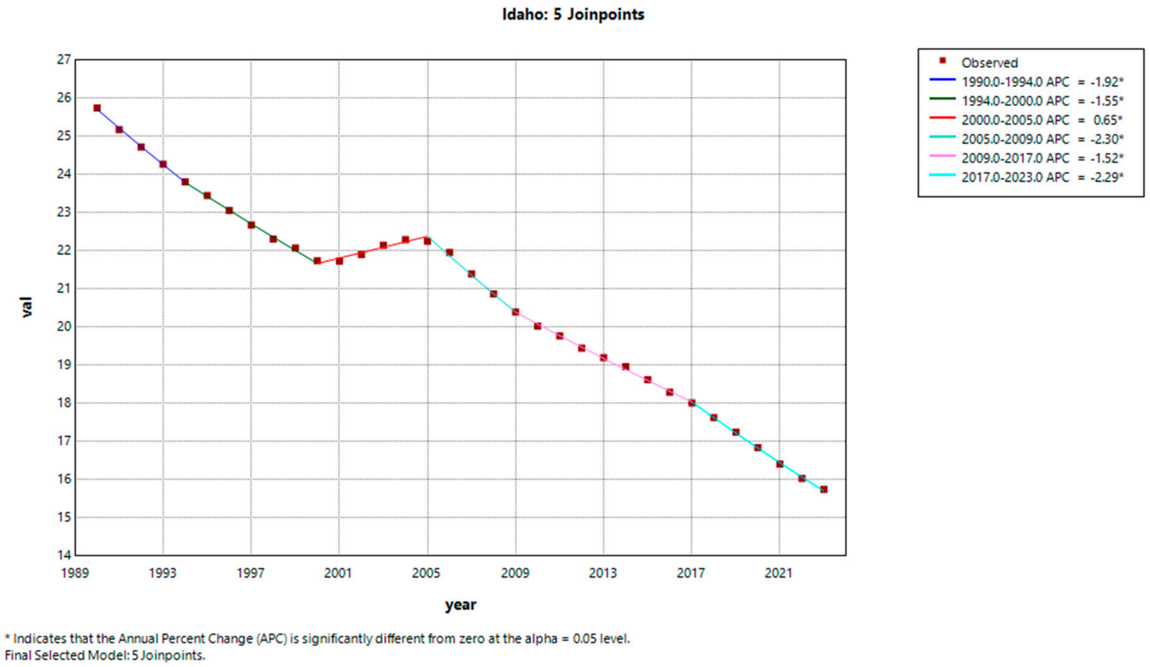

Figure S14. Joinpoint analysis of IDID attributable to Pb exposure in Illinois from 1990 to 2023

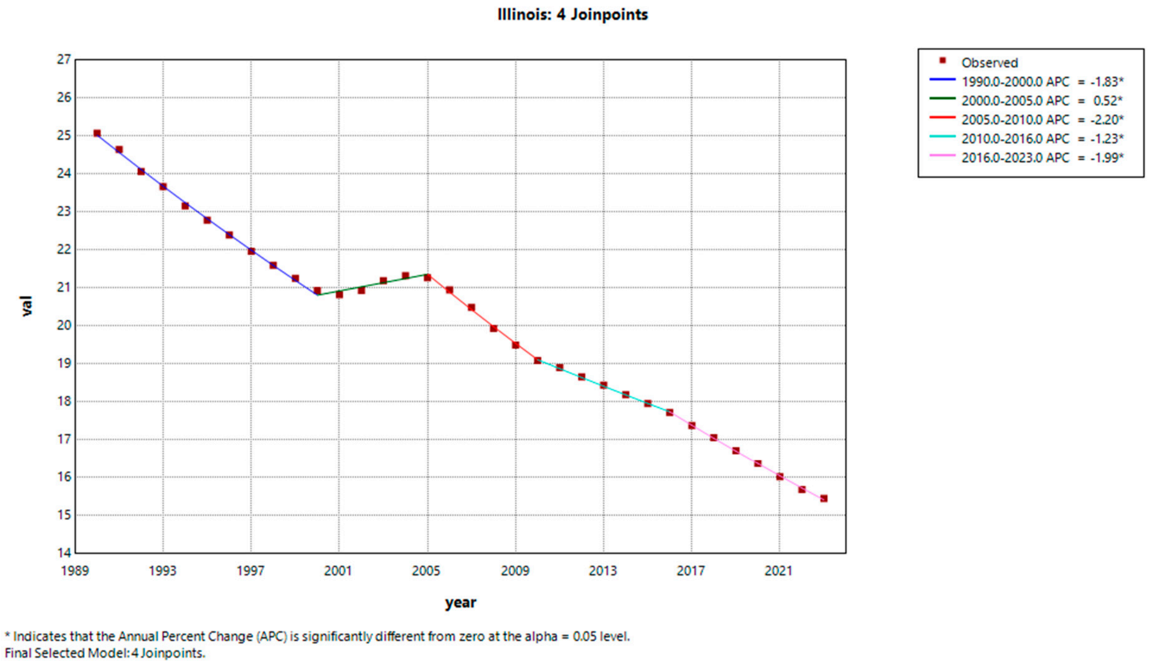

Figure S15. Joinpoint analysis of IDID attributable to Pb exposure in Indiana from 1990 to 2023

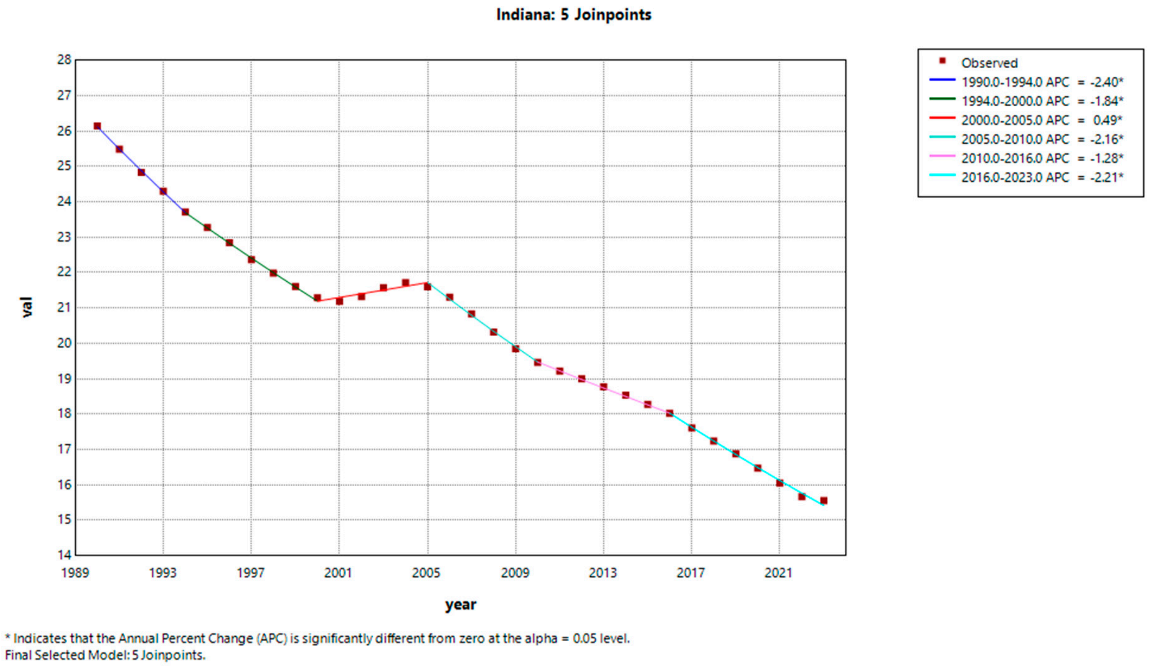

Figure S16. Joinpoint analysis of IDID attributable to Pb exposure in Iowa from 1990 to 2023

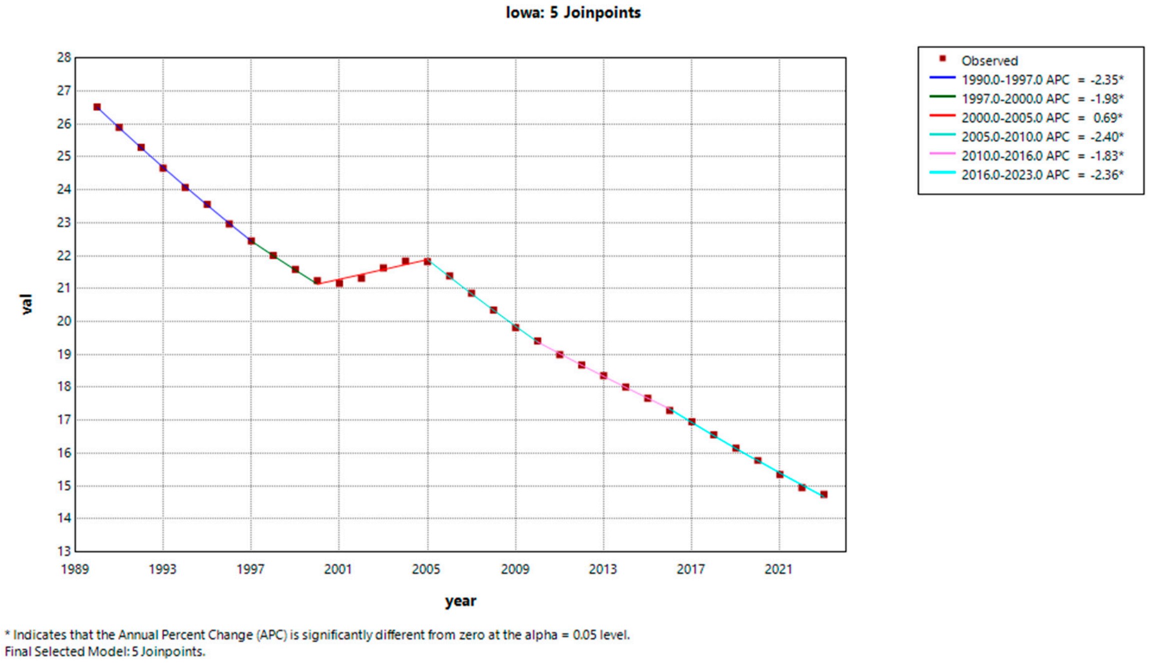

Figure S17. Joinpoint analysis of IDID attributable to Pb exposure in Kansas from 1990 to 2023

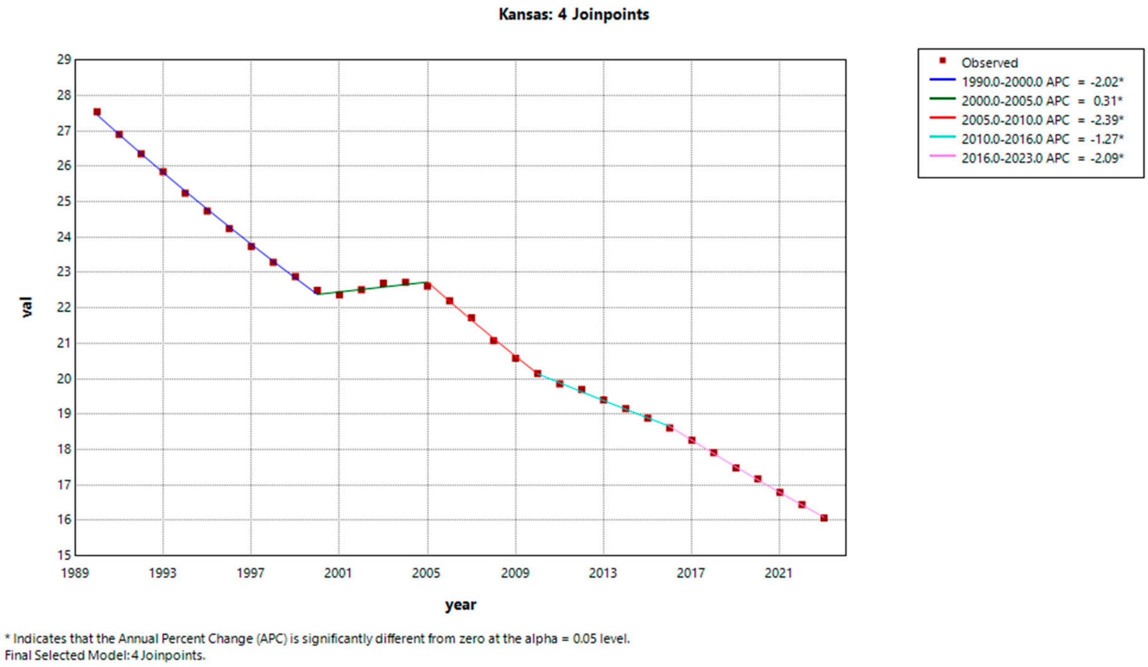

Figure S18. Joinpoint analysis of IDID attributable to Pb exposure in Kentucky from 1990 to 2023

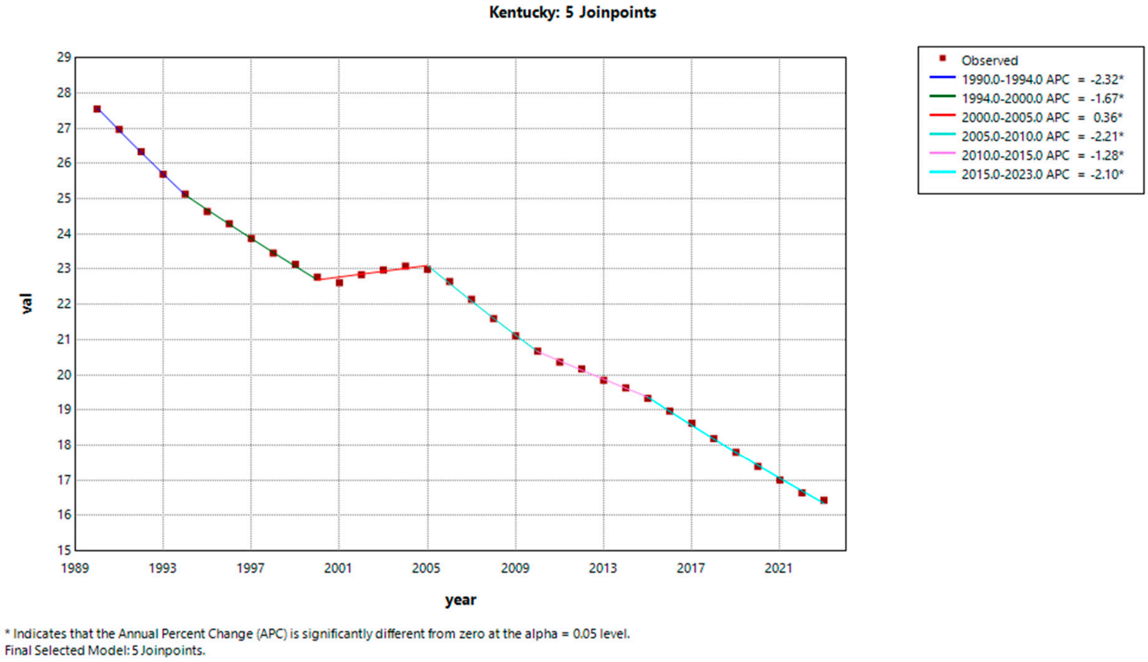

Figure S19. Joinpoint analysis of IDID attributable to Pb exposure in Louisiana from 1990 to 2023

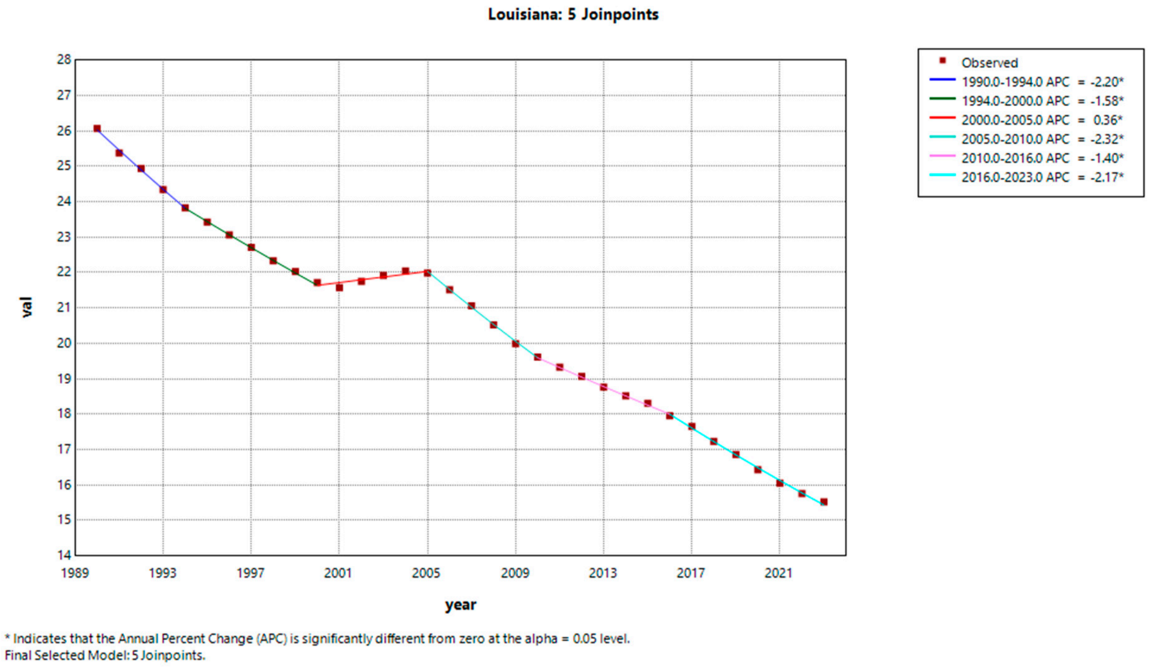

Figure S20. Joinpoint analysis of IDID attributable to Pb exposure in Maine from 1990 to 2023

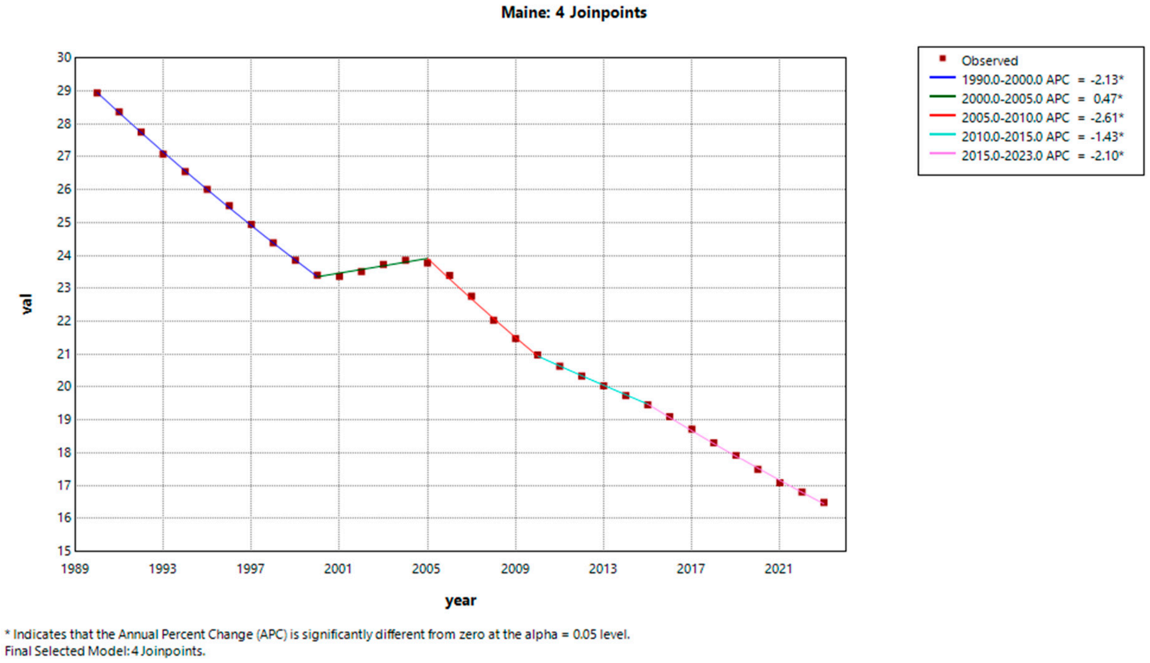

Figure S21. Joinpoint analysis of IDID attributable to Pb exposure in Maryland from 1990 to 2023

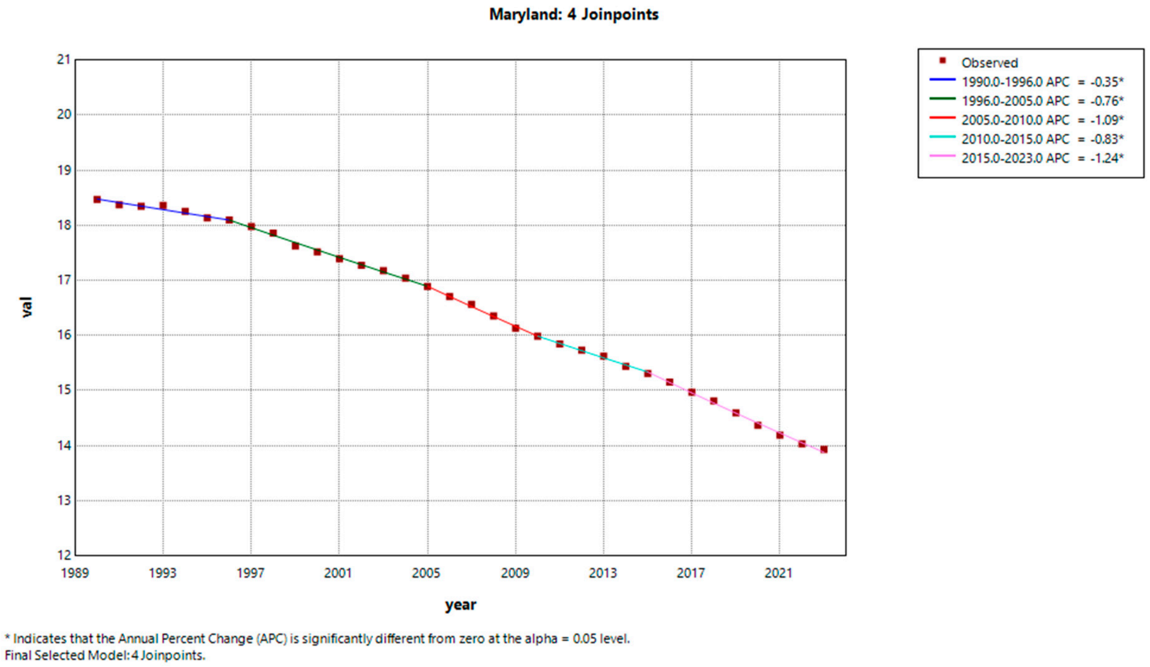

Figure S22. Joinpoint analysis of IDID attributable to Pb exposure in Massachusetts from 1990 to 2023

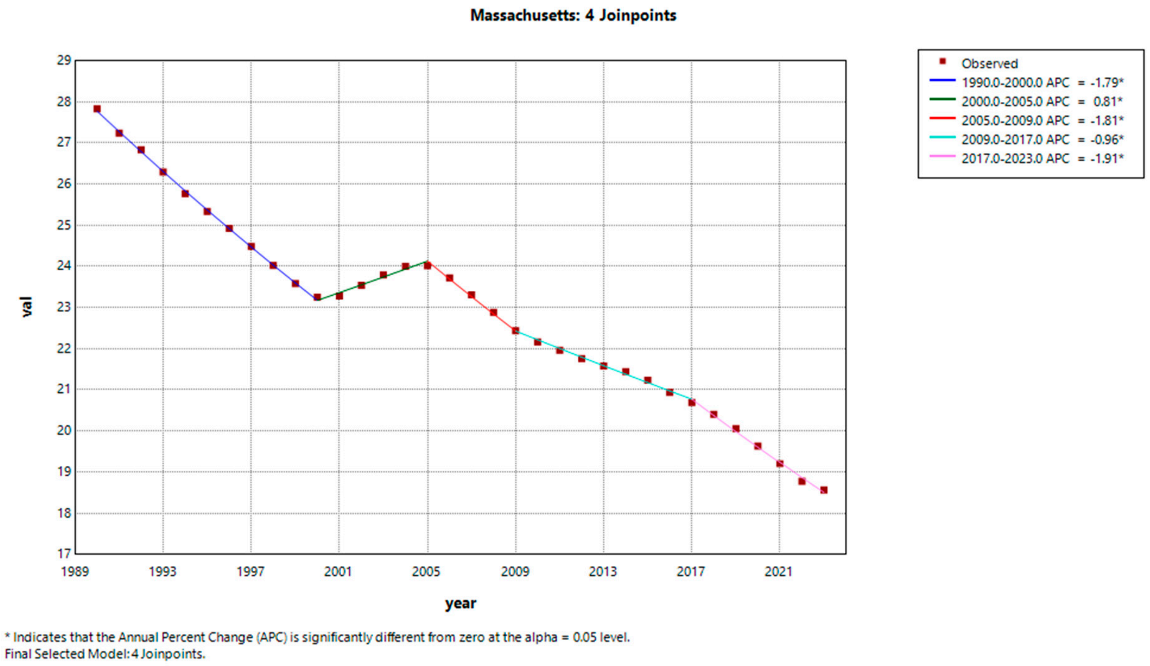

Figure S23. Joinpoint analysis of IDID attributable to Pb exposure in Michigan from 1990 to 2023

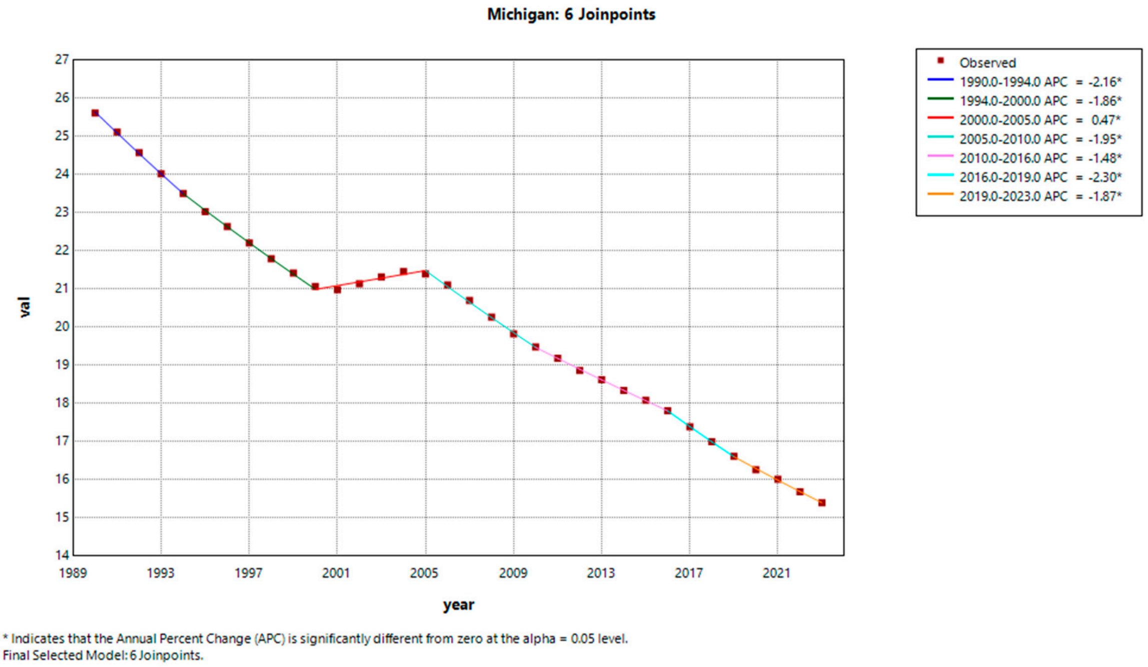

Figure S24. Joinpoint analysis of IDID attributable to Pb exposure in Minnesota from 1990 to 2023

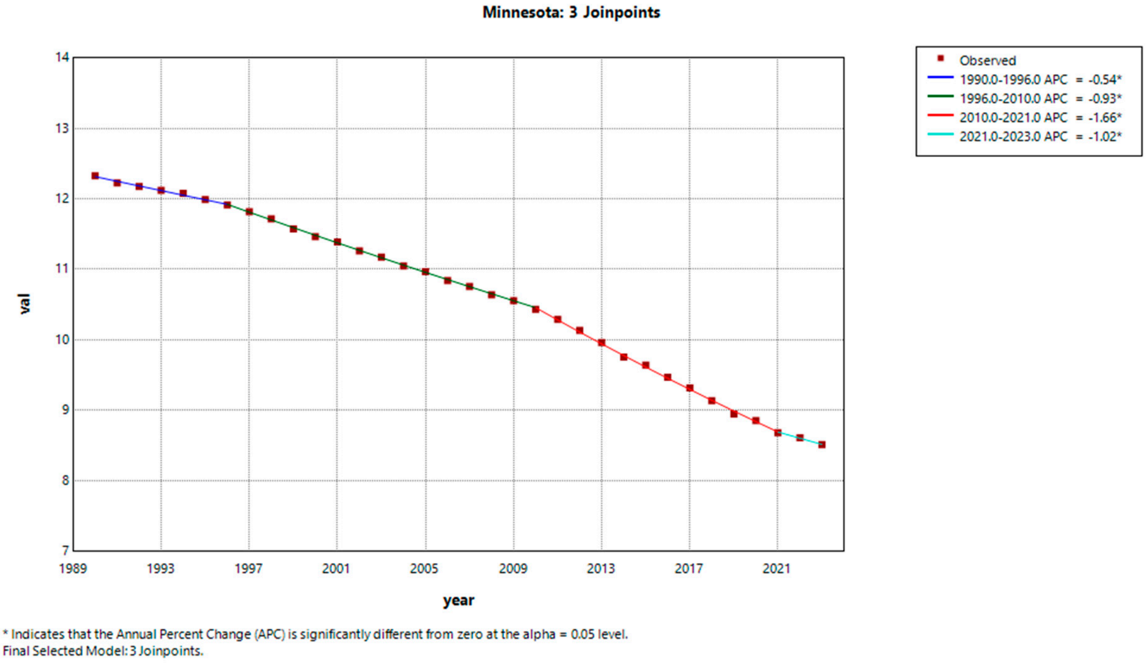

Figure S25. Joinpoint analysis of IDID attributable to Pb exposure in Mississippi from 1990 to 2023

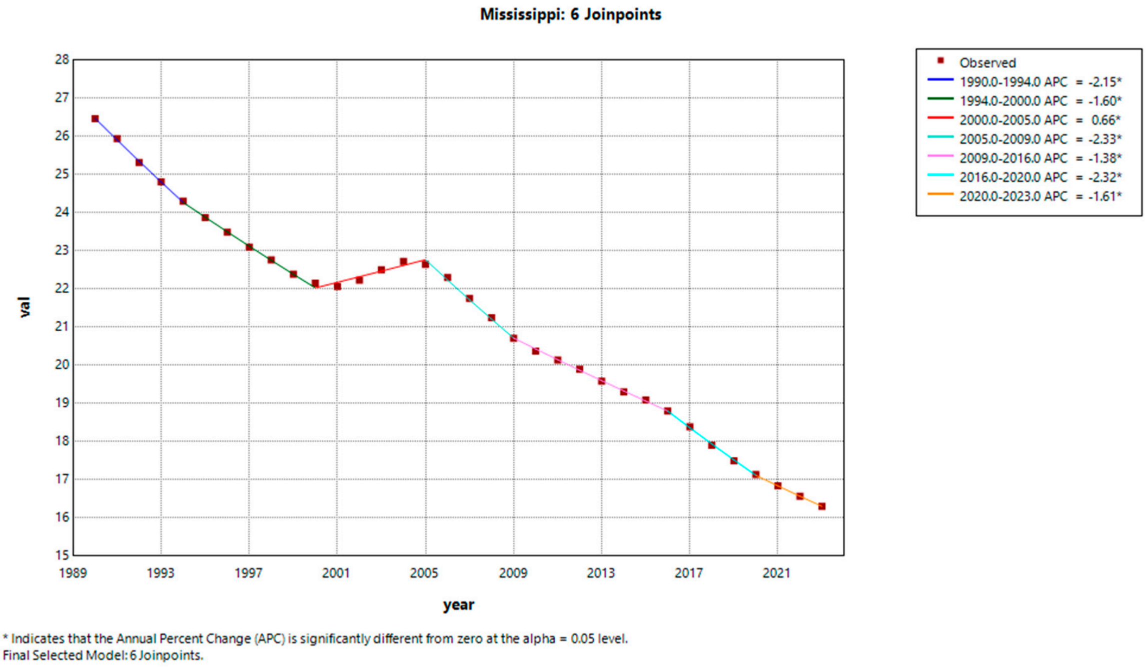

Figure S26. Joinpoint analysis of IDID attributable to Pb exposure in Missouri from 1990 to 2023

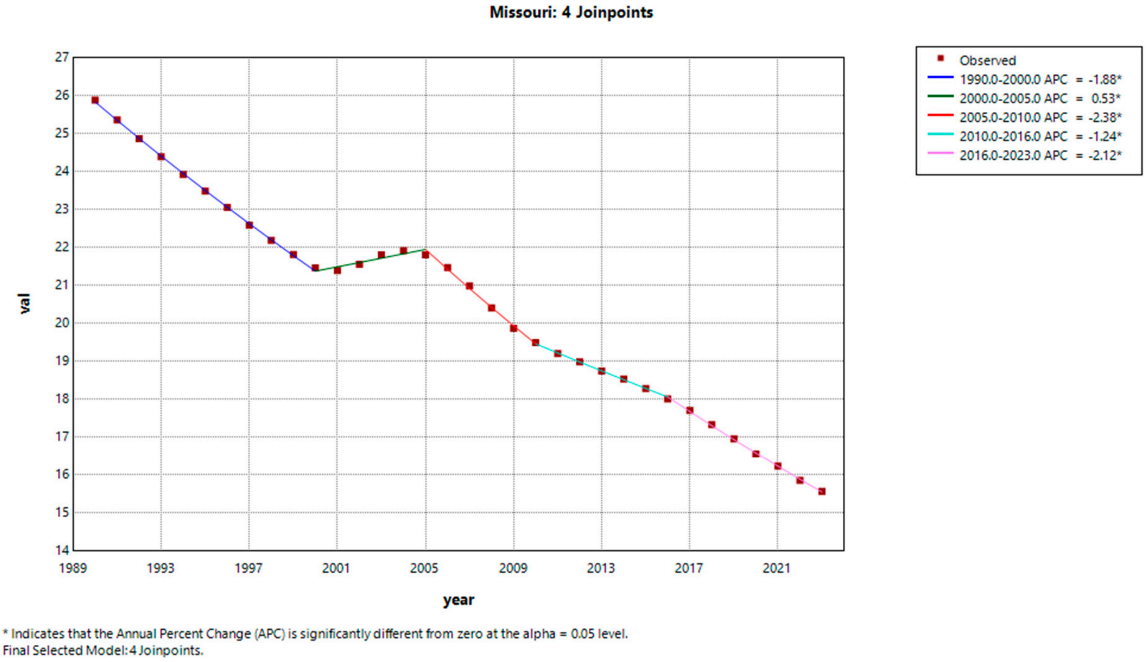

Figure S27. Joinpoint analysis of IDID attributable to Pb exposure in Montana from 1990 to 2023

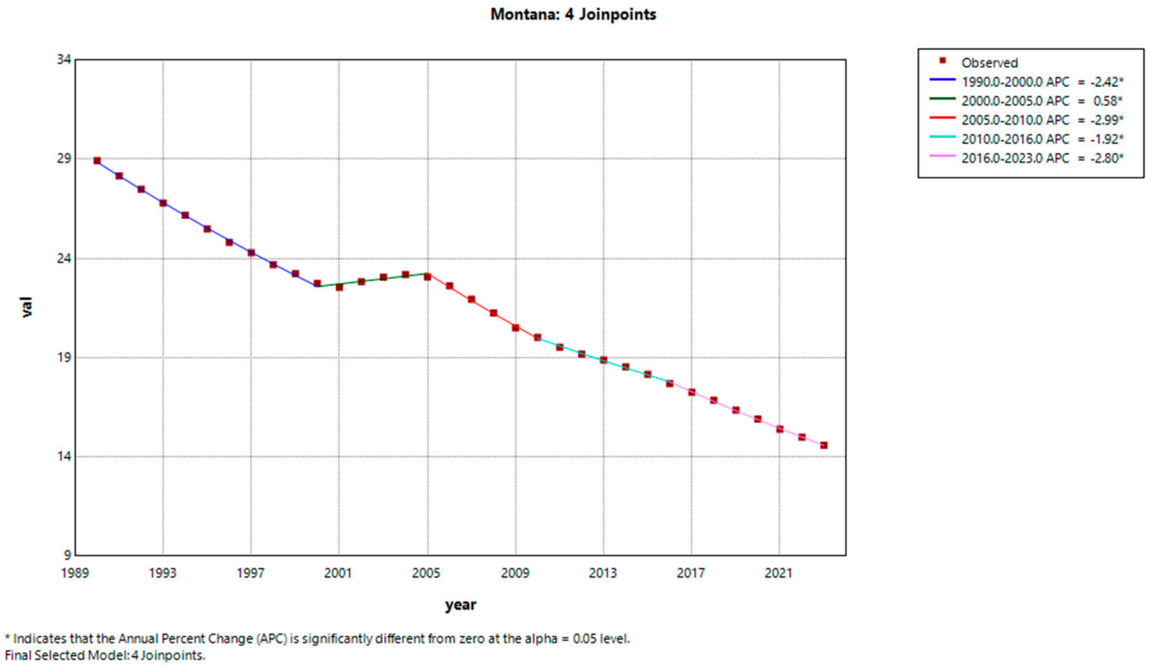

Figure S28. Joinpoint analysis of IDID attributable to Pb exposure in Nebraska from 1990 to 2023

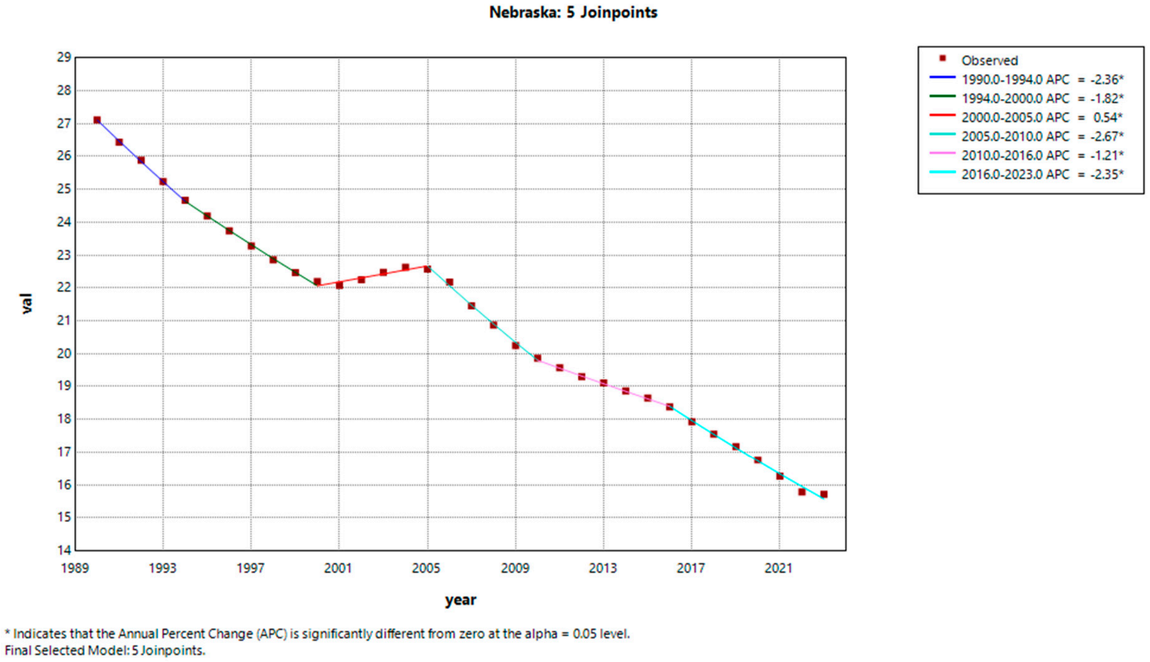

Figure S29. Joinpoint analysis of IDID attributable to Pb exposure in Navada from 1990 to 2023

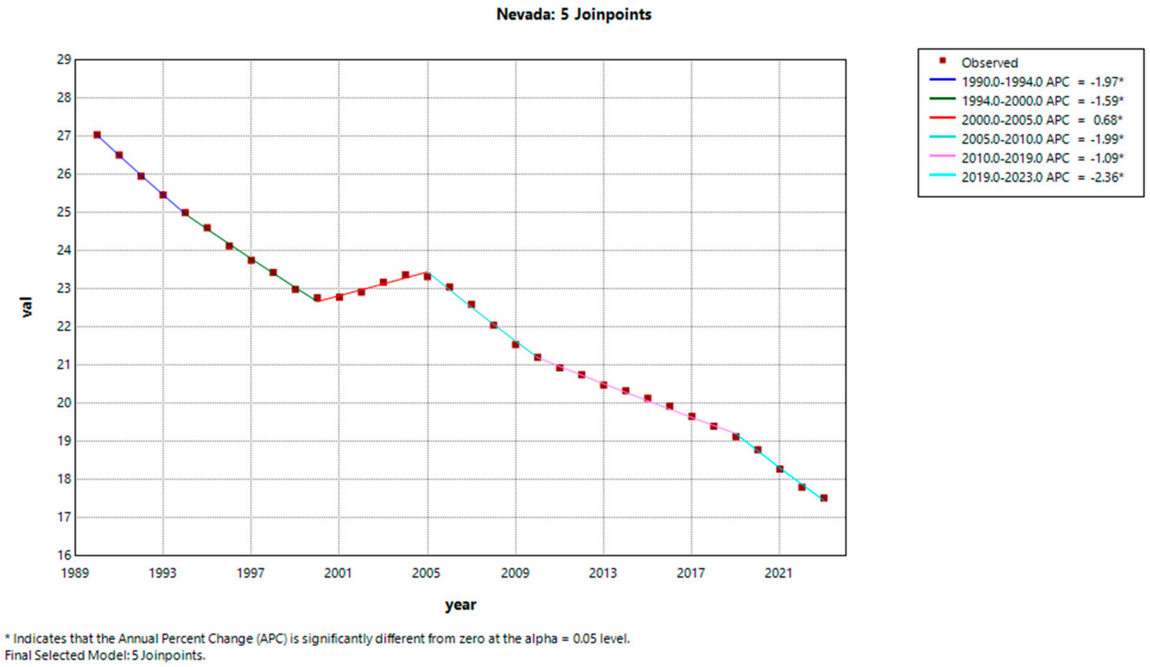

Figure S30. Joinpoint analysis of IDID attributable to Pb exposure in New Hampshire from 1990 to 2023

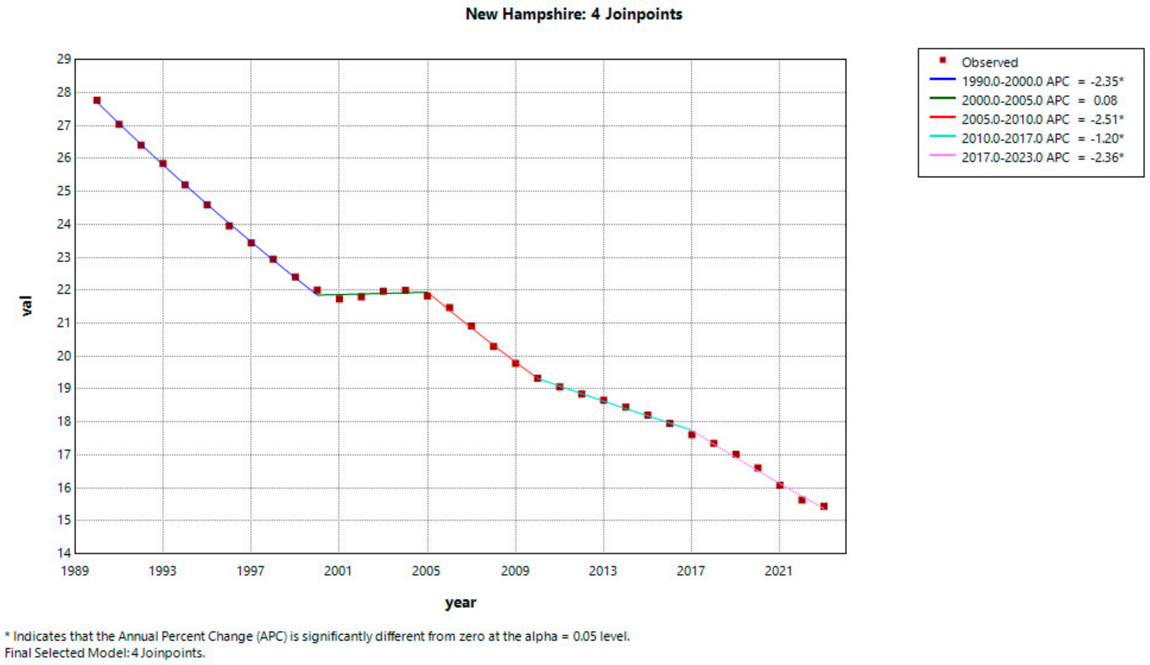

Figure S31. Joinpoint analysis of IDID attributable to Pb exposure in New Jersey from 1990 to 2023

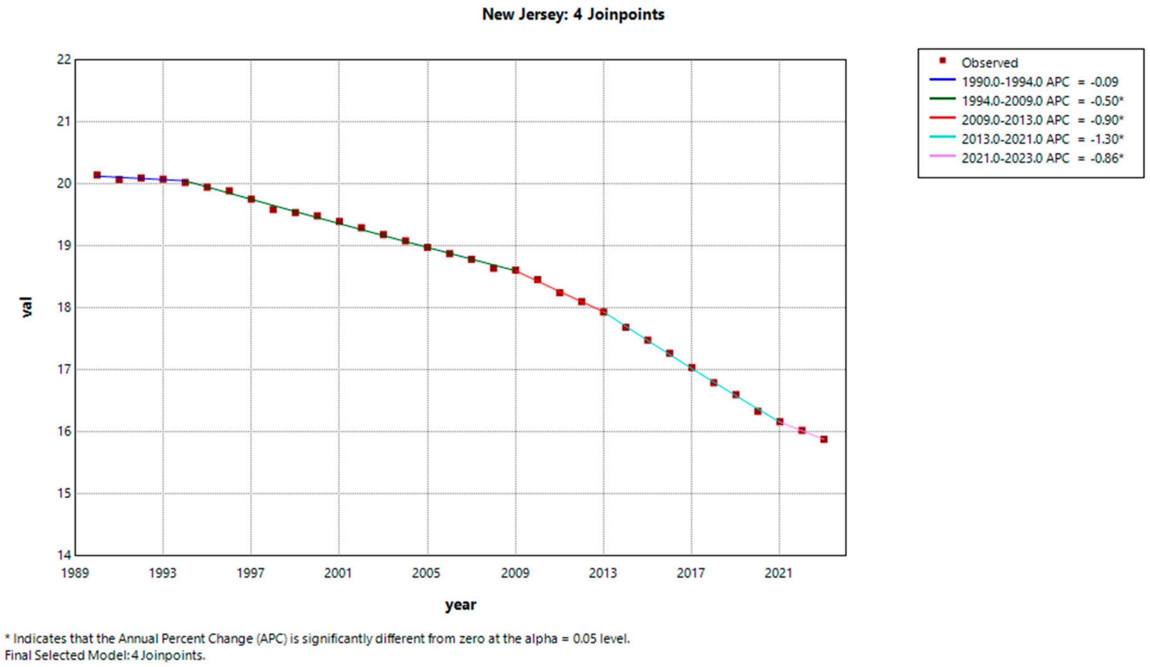

Figure S32. Joinpoint analysis of IDID attributable to Pb exposure in New Mexico from 1990 to 2023

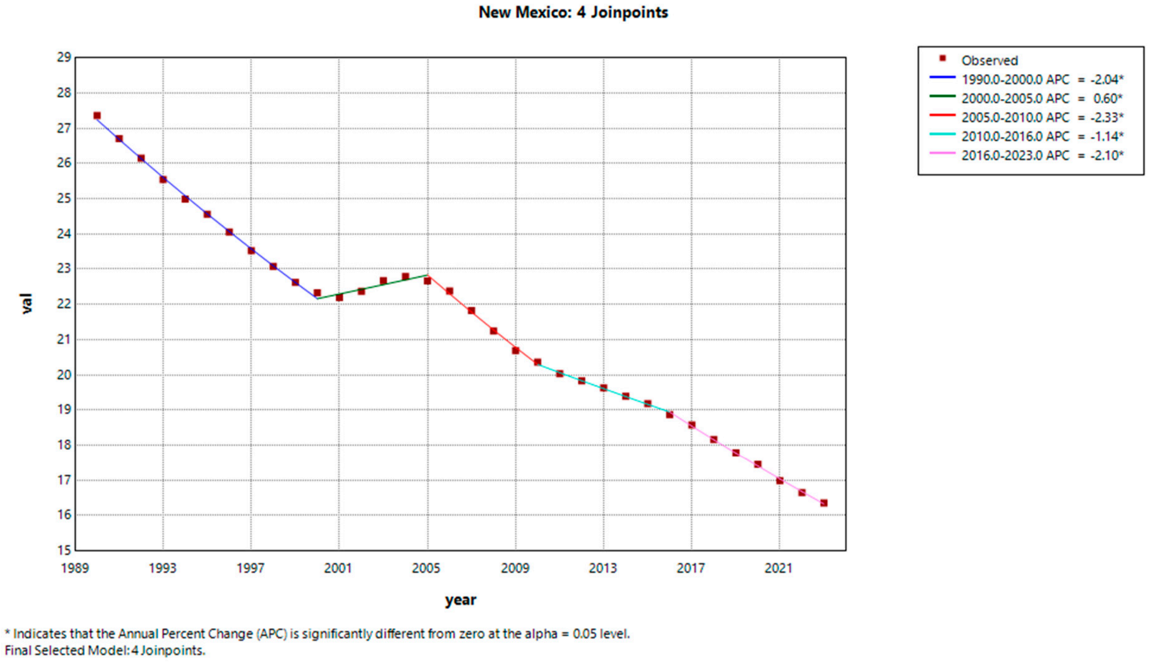

Figure S33. Joinpoint analysis of IDID attributable to Pb exposure in New York from 1990 to 2023

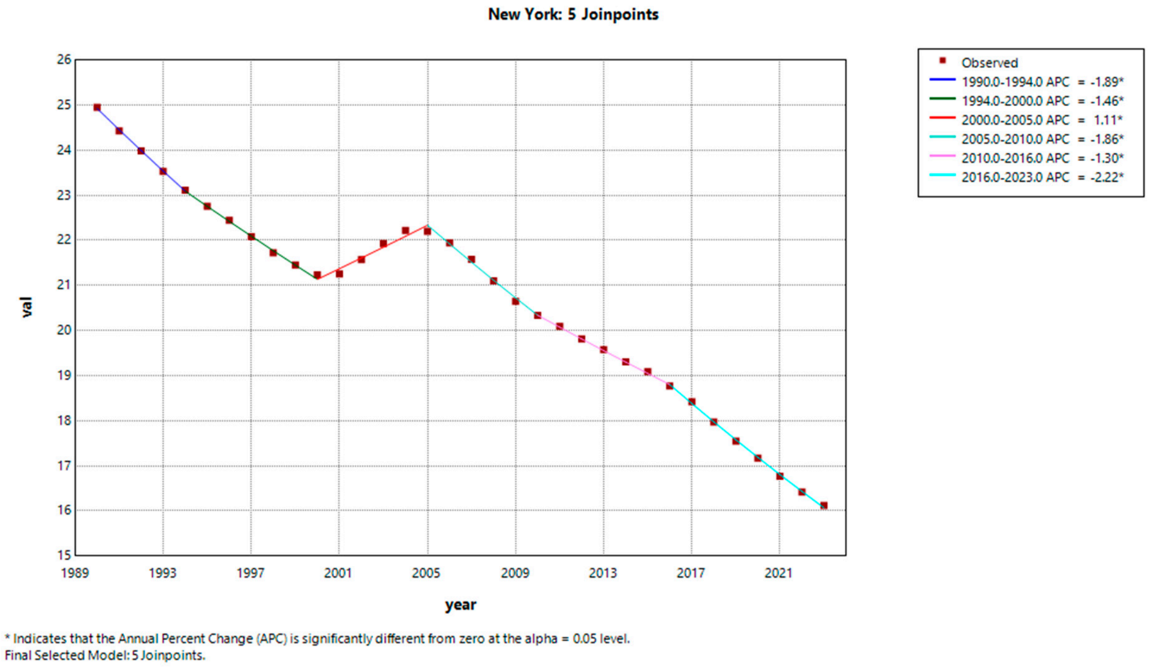

Figure S34. Joinpoint analysis of IDID attributable to Pb exposure in North Carolina from 1990 to 2023

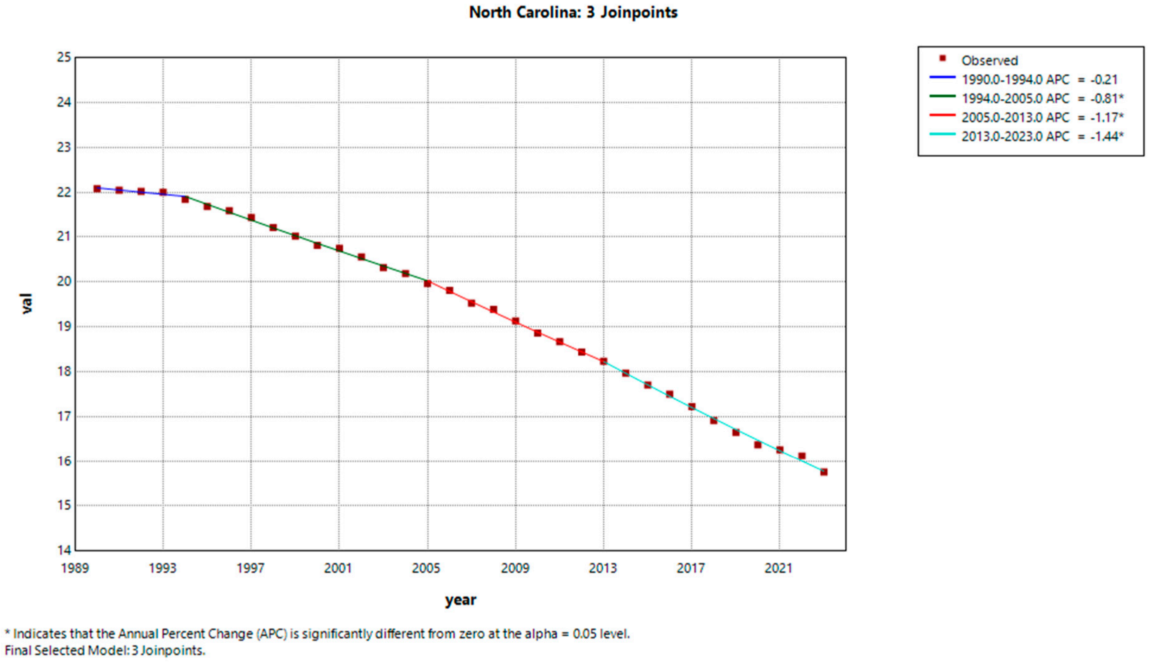

Figure S35. Joinpoint analysis of IDID attributable to Pb exposure in North Dakota from 1990 to 2023

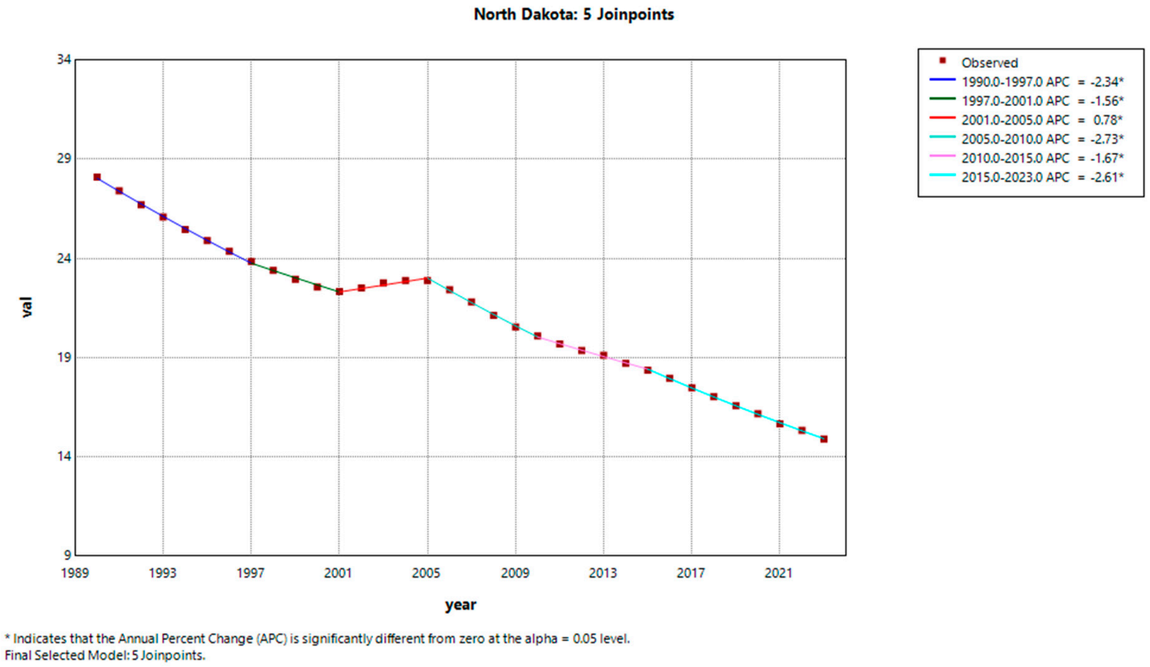

Figure S36. Joinpoint analysis of IDID attributable to Pb exposure in Ohio from 1990 to 2023

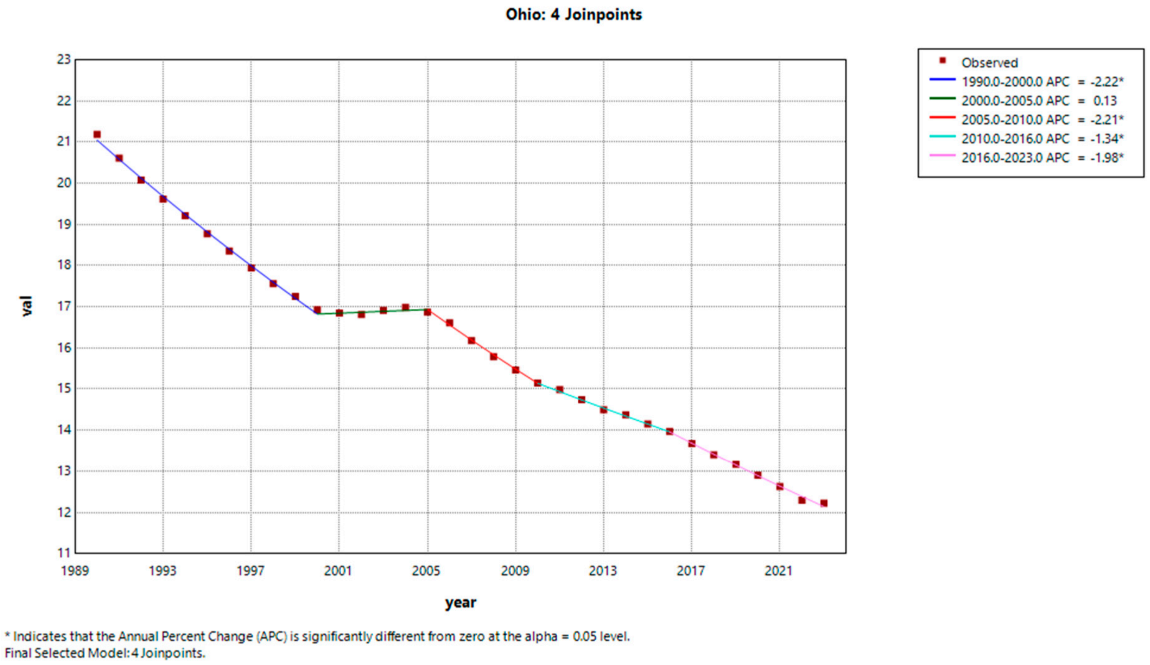

Figure S37. Joinpoint analysis of IDID attributable to Pb exposure in Oklahoma from 1990 to 2023

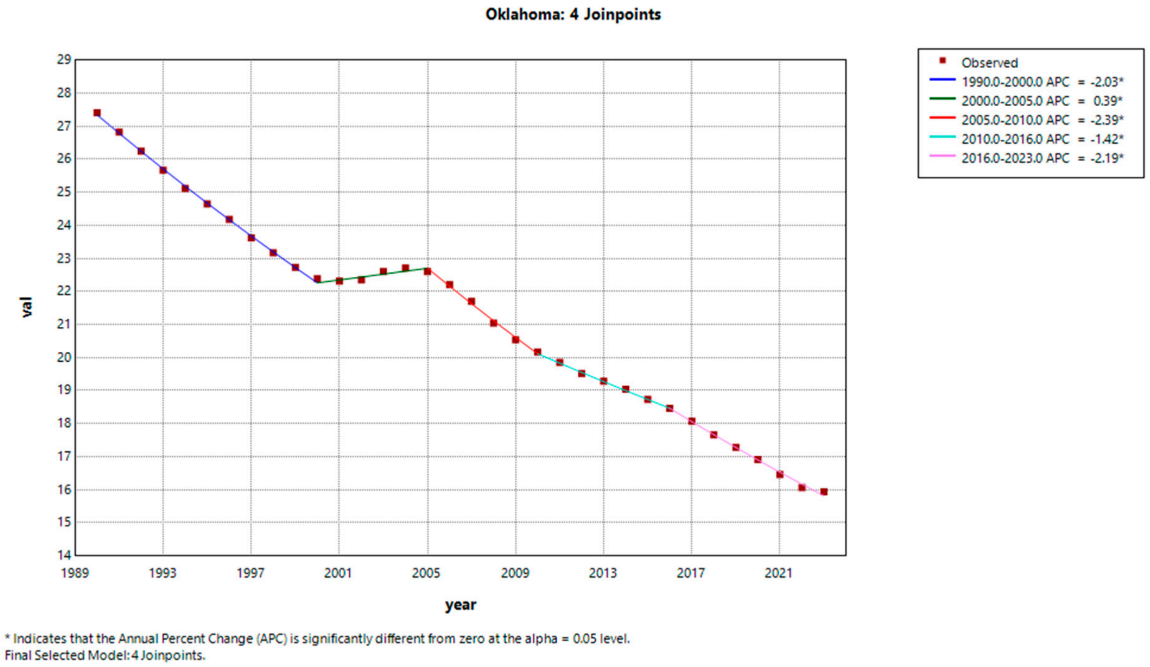

Figure S38. Joinpoint analysis of IDID attributable to Pb exposure in Oregon from 1990 to 2023

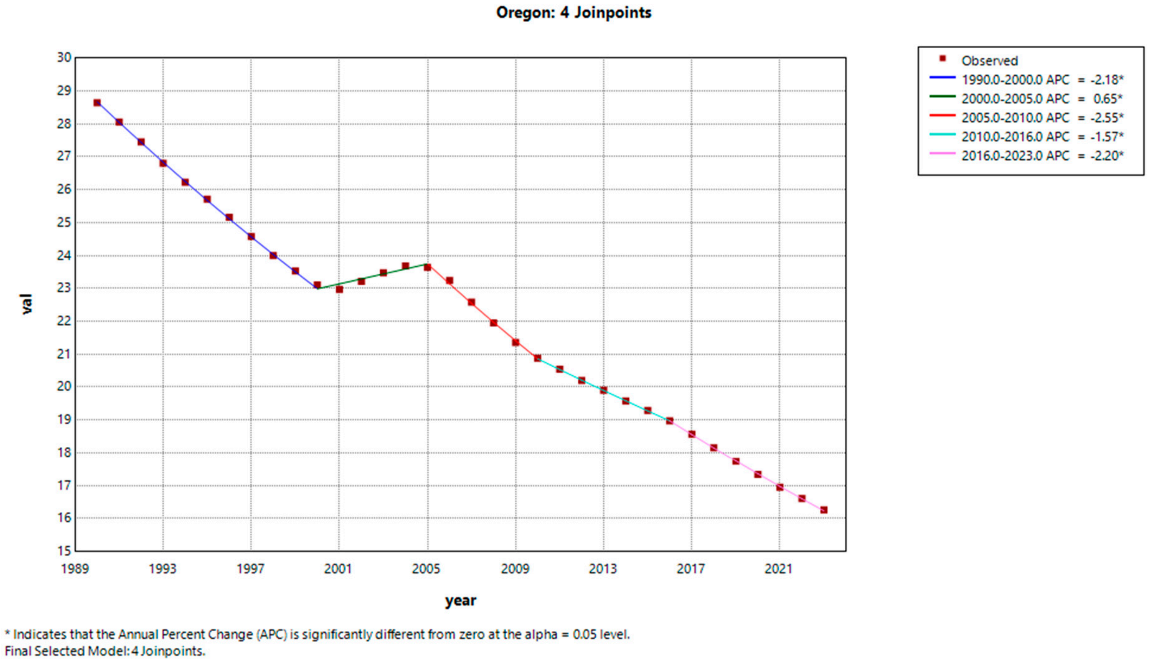

Figure S39. Joinpoint analysis of IDID attributable to Pb exposure in Pennsylvania from 1990 to 2023

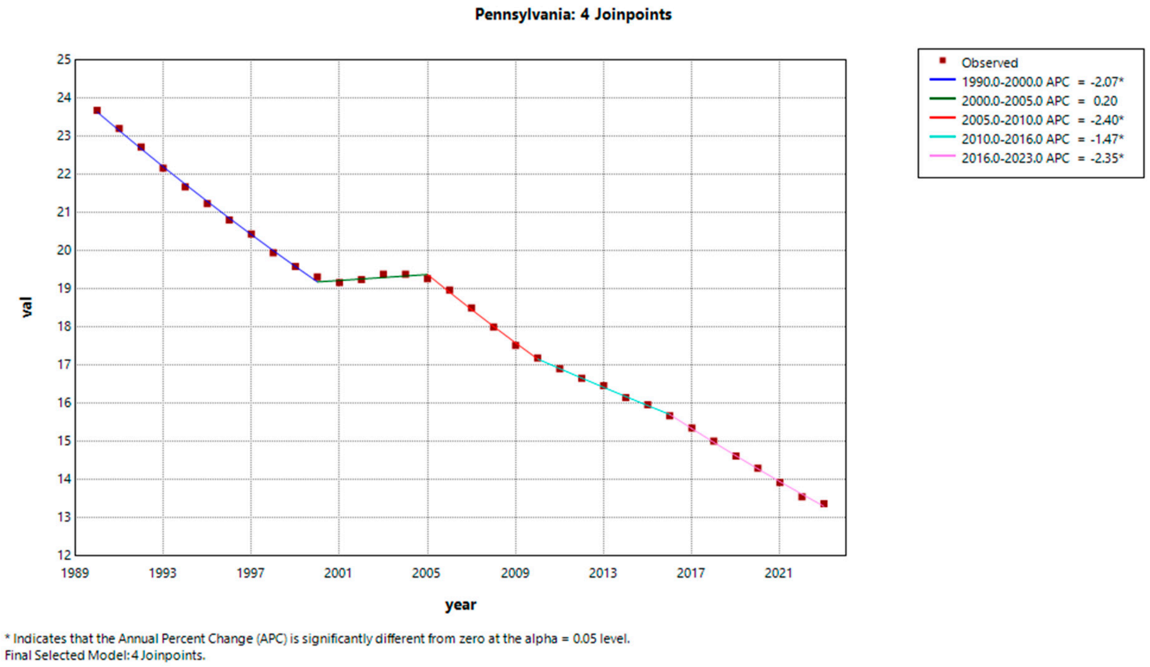

Figure S40. Joinpoint analysis of IDID attributable to Pb exposure in Rhode Island from 1990 to 2023

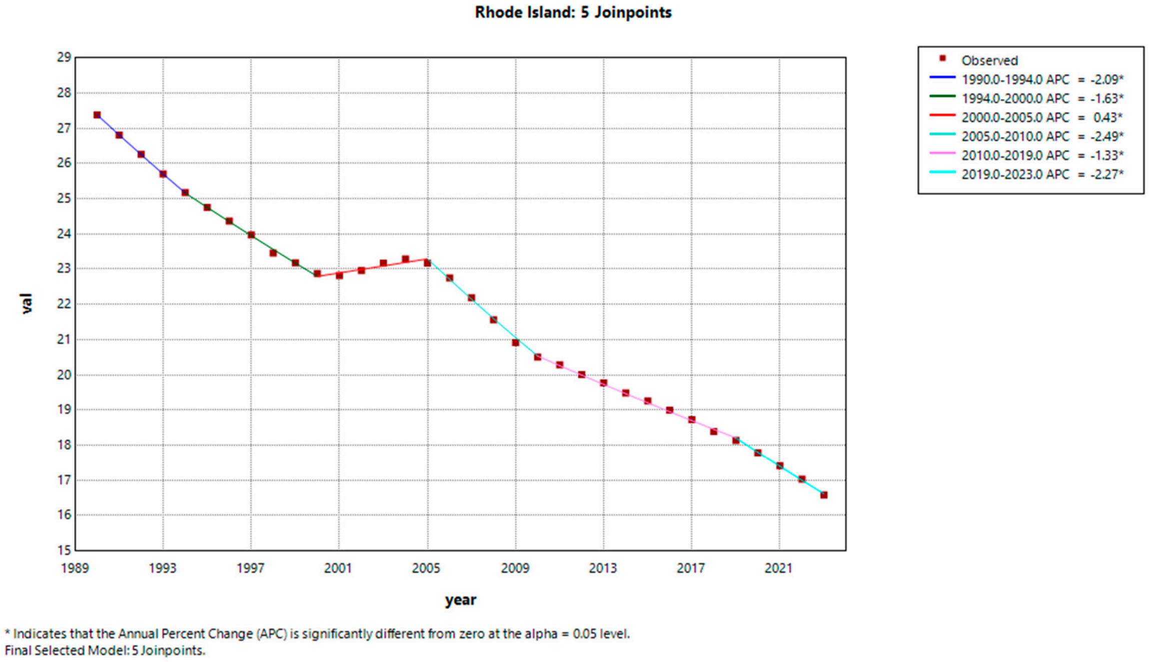

Figure S41. Joinpoint analysis of IDID attributable to Pb exposure in South Carolina from 1990 to 2023

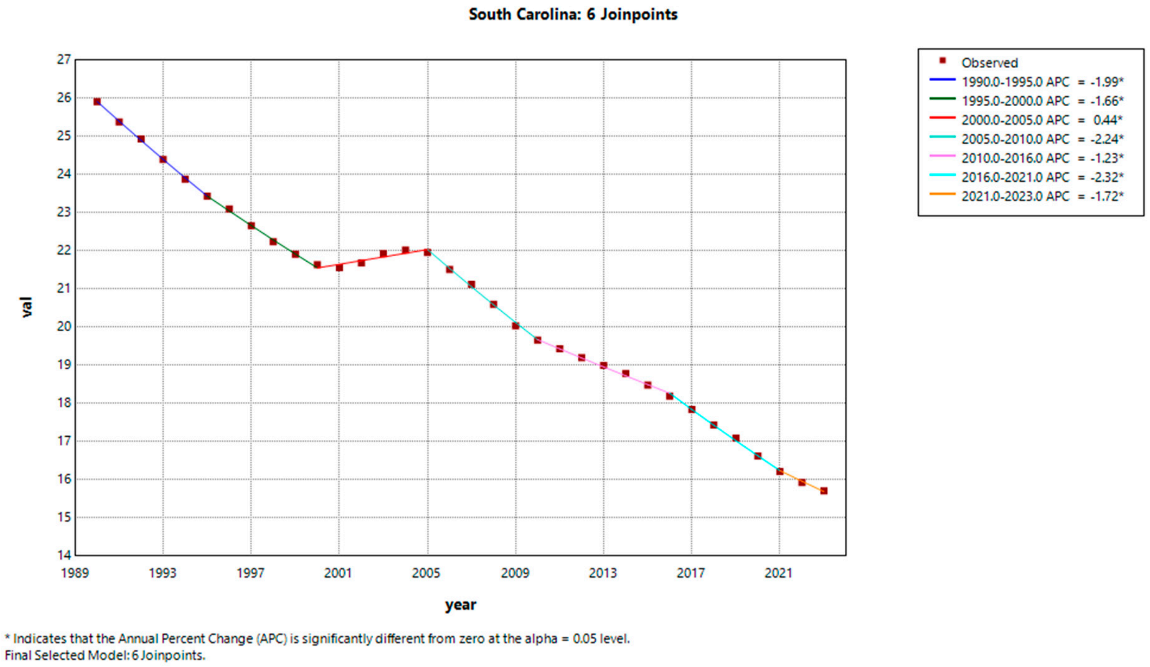

Figure S42. Joinpoint analysis of IDID attributable to Pb exposure in South Dakota from 1990 to 2023

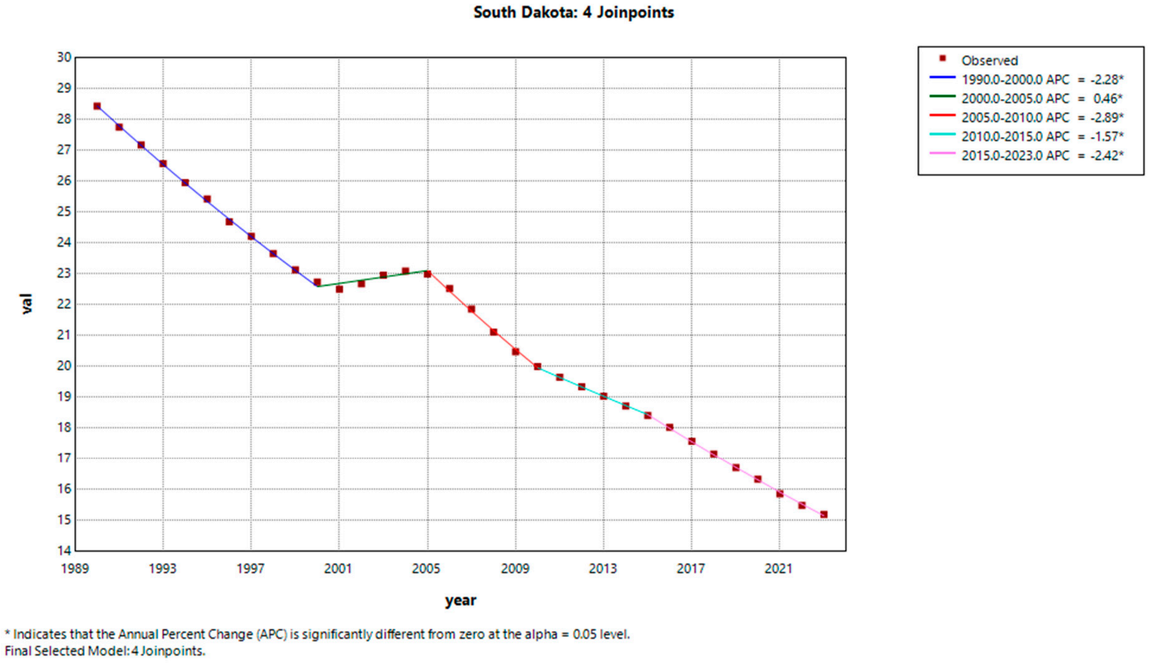

Figure S43. Joinpoint analysis of IDID attributable to Pb exposure in Tennessee from 1990 to 2023

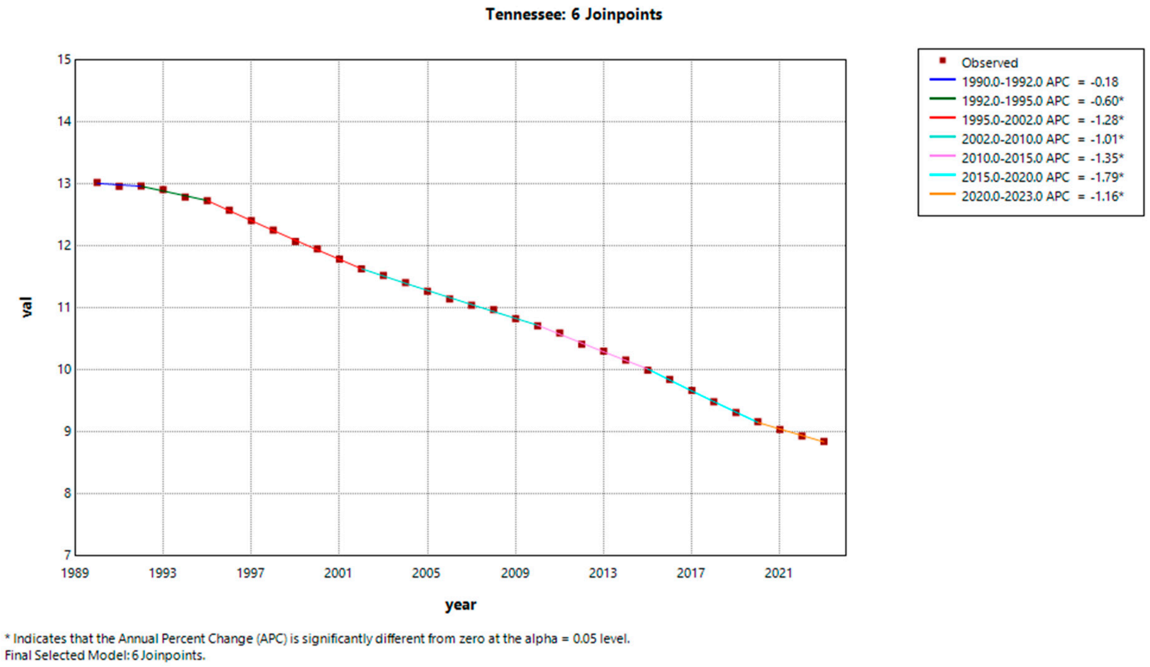

Figure S44. Joinpoint analysis of IDID attributable to Pb exposure in Texas from 1990 to 2023

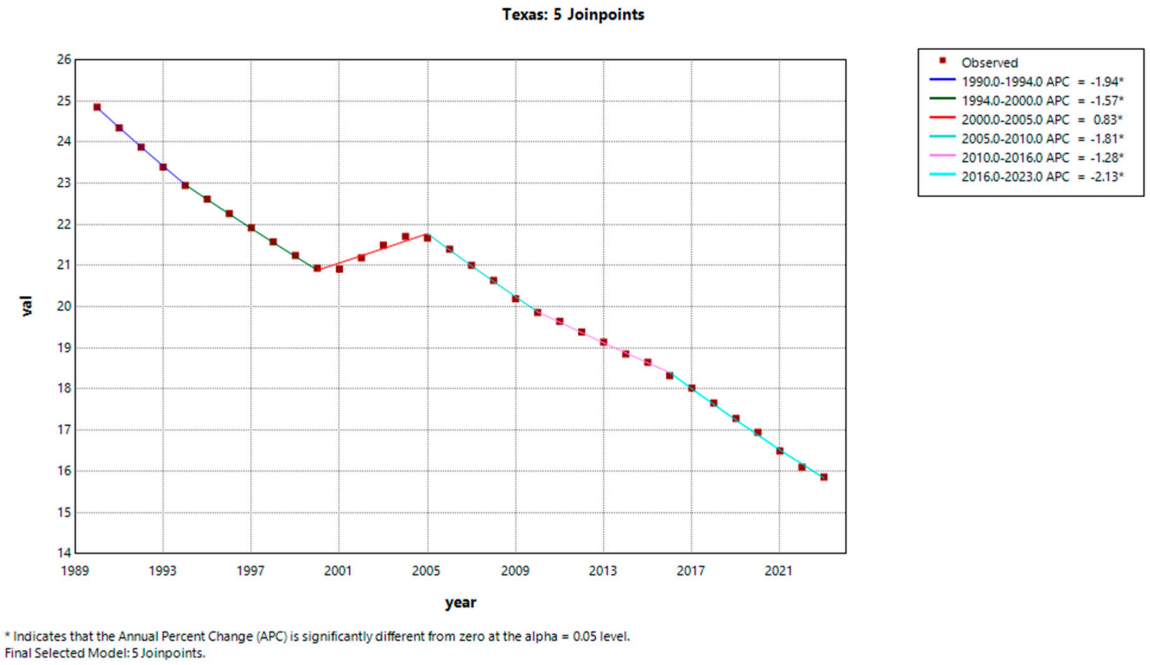

Figure S45. Joinpoint analysis of IDID attributable to Pb exposure in Utah from 1990 to 2023

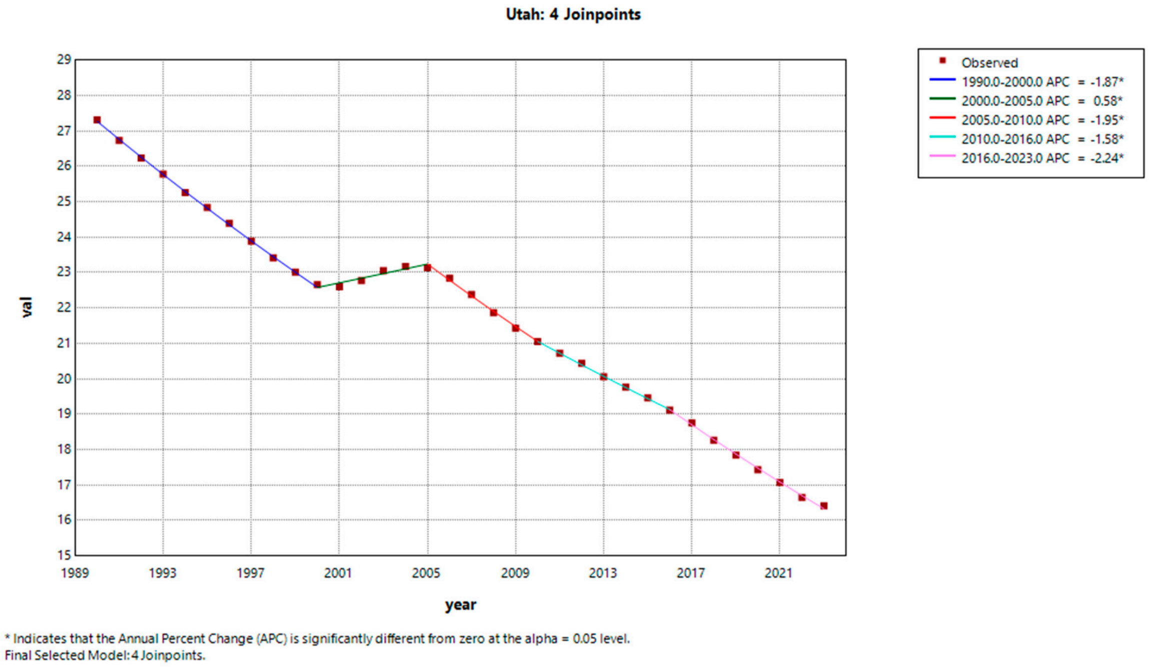

Figure S46. Joinpoint analysis of IDID attributable to Pb exposure in Vermont from 1990 to 2023

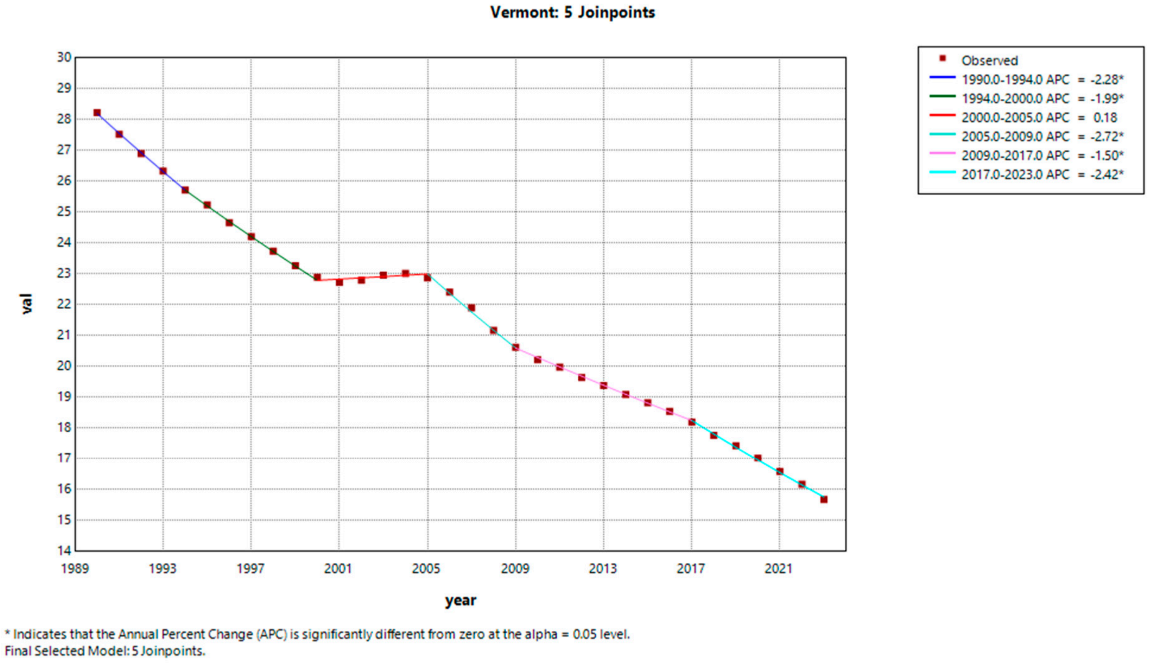

Figure S47. Joinpoint analysis of IDID attributable to Pb exposure in Virginia from 1990 to 2023

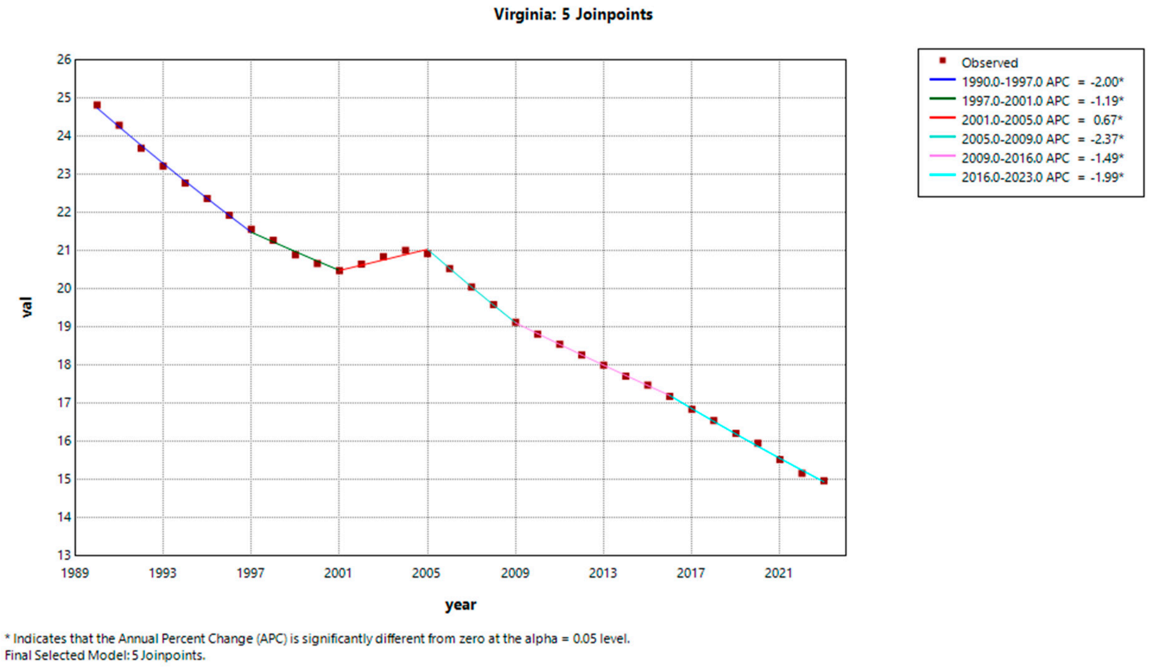

Figure S48. Joinpoint analysis of IDID attributable to Pb exposure in Washington from 1990 to 2023

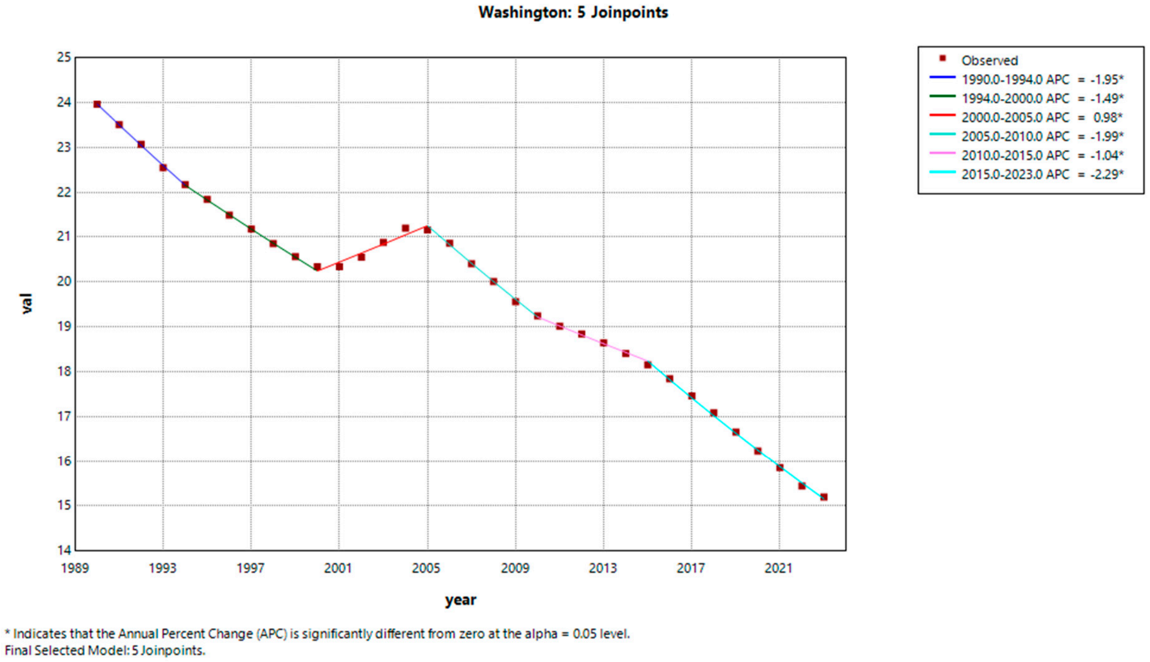

Figure S49. Joinpoint analysis of IDID attributable to Pb exposure in West Virginia from 1990 to 2023

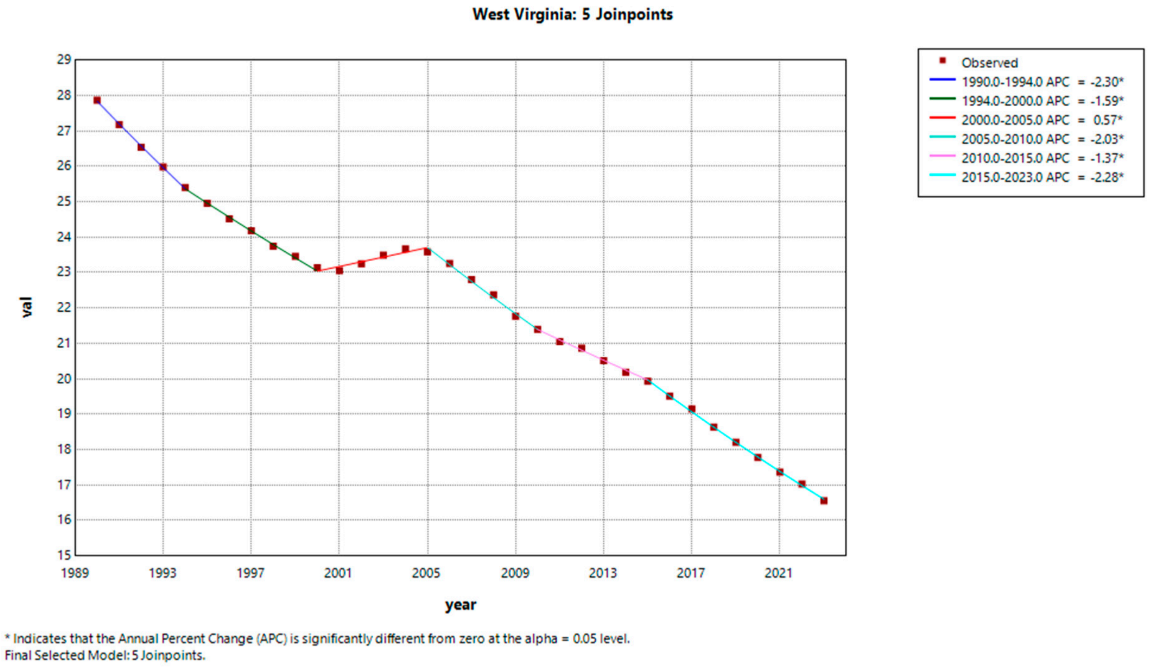

Figure S50. Joinpoint analysis of IDID attributable to Pb exposure in Wisconsin from 1990 to 2023

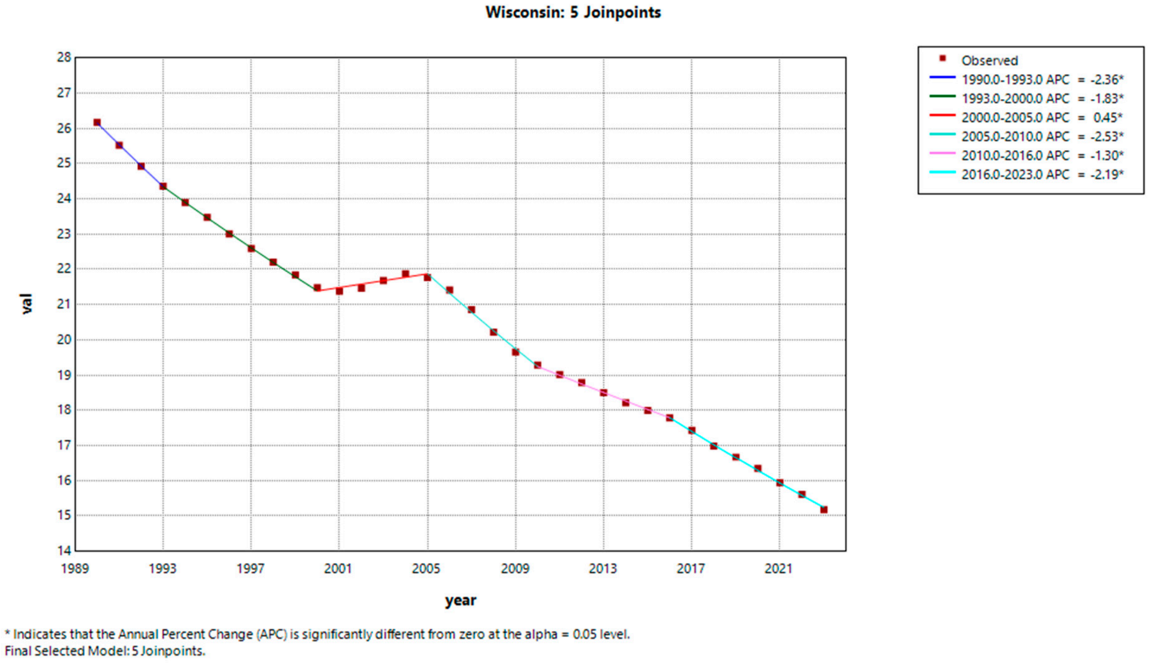

Figure S51. Joinpoint analysis of IDID attributable to Pb exposure in Wyoming from 1990 to 2023

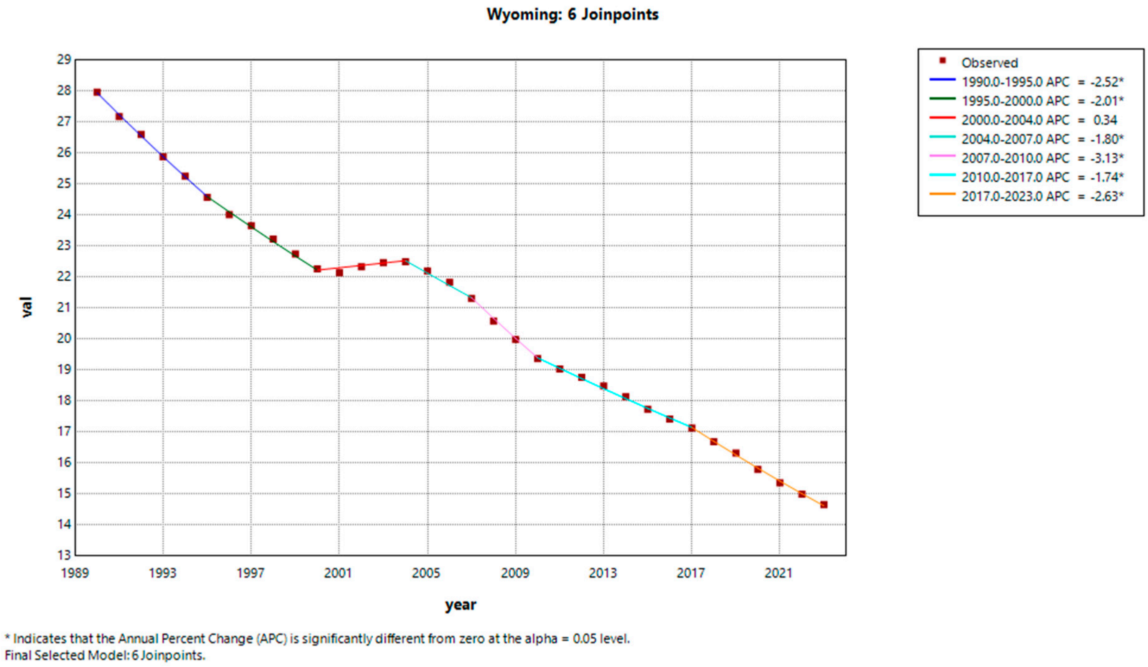

Figure S52. Observed and predicted DALYs in females from the backcasting analysis (2019-2023).

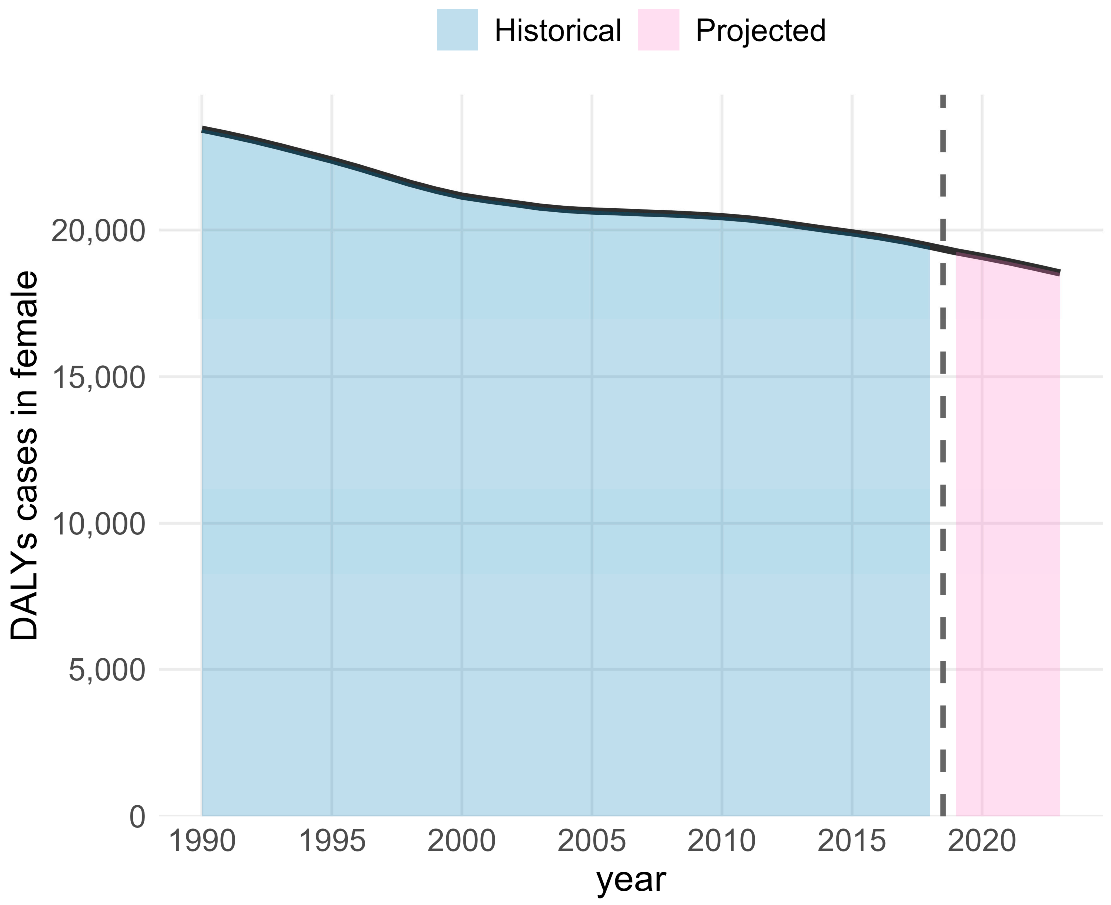

Figure S53. Observed and predicted DALYs in males from the backcasting analysis (2019-2023).

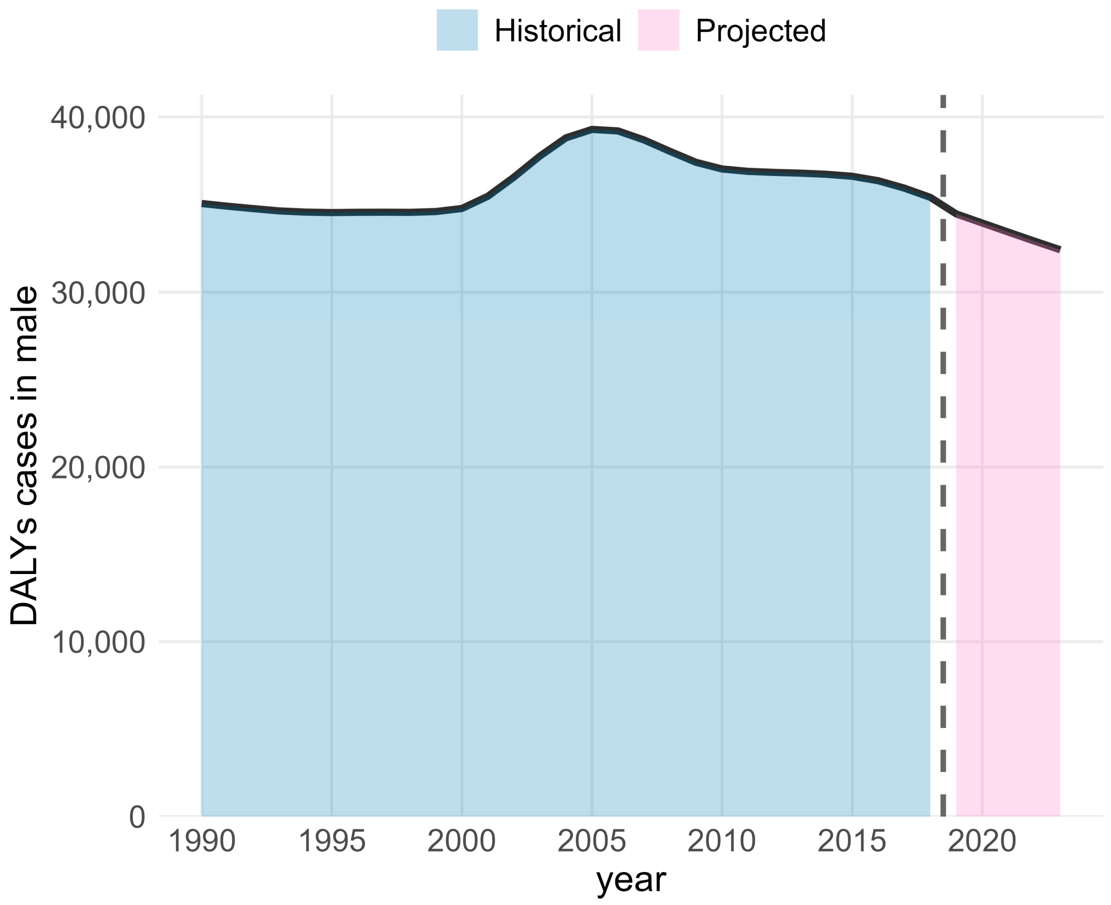

Figure S54. Observed and predicted ASDR in females from the backcasting analysis (2019-2023).

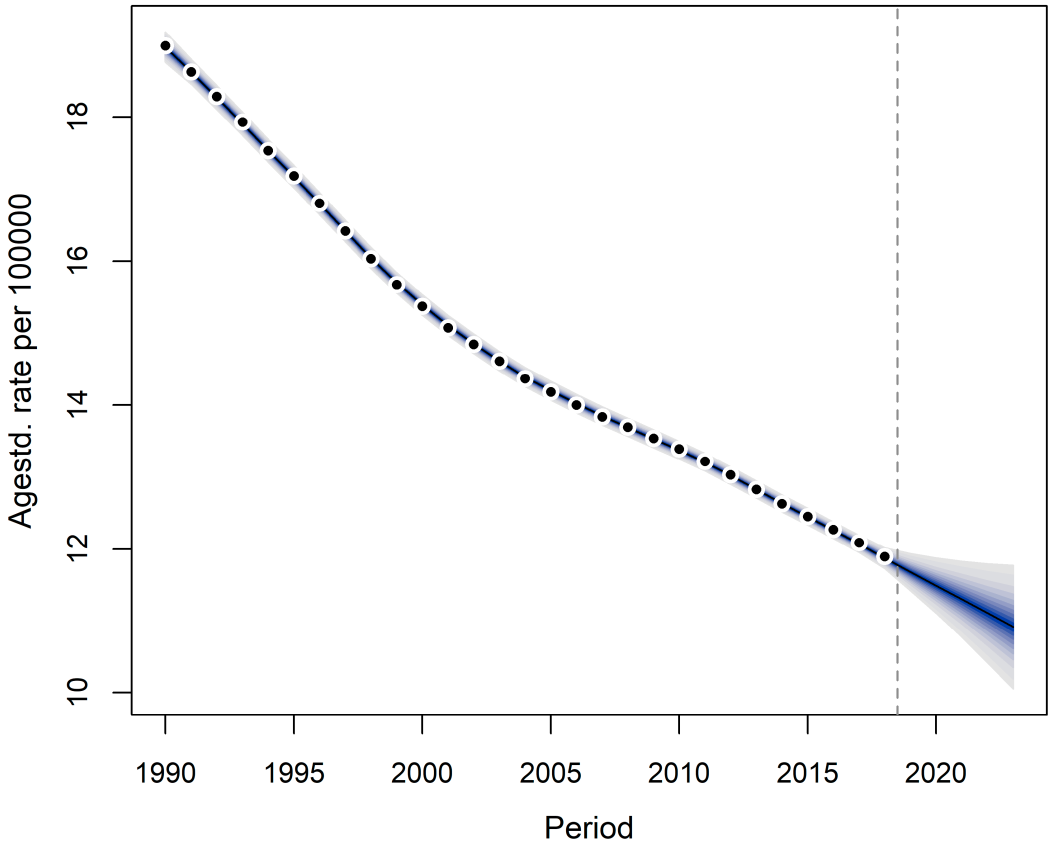

Figure S55. Observed and predicted ASDR in males from the backcasting analysis (2019-2023).

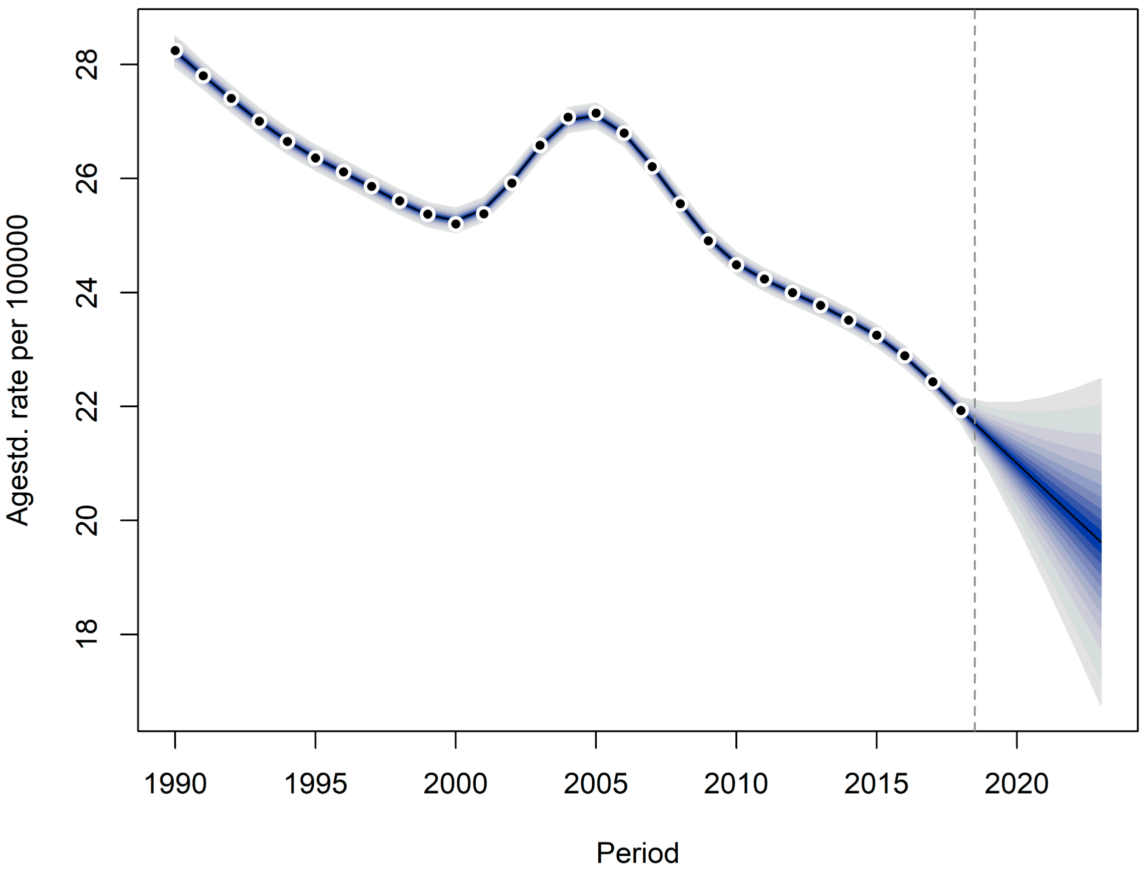

28
26
24
22
20
18
Agestd. rate per 100000
1990
1995
2000
2005
2010
2015
2020
Period
